# Peripheral neuronal activation shapes the microbiome and alters gut physiology

**DOI:** 10.1016/j.celrep.2024.113953

**Published:** 2024-03-21

**Authors:** Jessica A. Griffiths, Bryan B. Yoo, Peter Thuy-Boun, Victor J. Cantu, Kelly C. Weldon, Collin Challis, Michael J. Sweredoski, Ken Y. Chan, Taren M. Thron, Gil Sharon, Annie Moradian, Gregory Humphrey, Qiyun Zhu, Justin P. Shaffer, Dennis W. Wolan, Pieter C. Dorrestein, Rob Knight, Viviana Gradinaru, Sarkis K. Mazmanian

**Affiliations:** 1 Division of Biology & Biological Engineering, California Institute of Technology, Pasadena, CA 91125, USA; 2 Aligning Science Across Parkinson’s (ASAP) Collaborative Research Network, Chevy Chase, MD 20815, USA; 3 Departments of Molecular Medicine and Integrative Structural and Computational Biology, The Scripps Research Institute, 10550 North Torrey Pines Road, La Jolla, CA 92037, USA; 4 Department of Pediatrics, University of California, San Diego, San Diego, CA, USA; 5 Collaborative Mass Spectrometry Innovation Center, Skaggs School of Pharmacy and Pharmaceutical Sciences, University of California, San Diego, San Diego, CA, USA; 6 UCSD Center for Microbiome Innovation, University of California, San Diego, San Diego, CA, USA; 7 Department of Computer Science and Engineering, University of California, San Diego, San Diego, CA, USA; 8 Shu Chien-Gene Lay Department of Engineering, University of California, San Diego, San Diego, CA, USA; 9 Halıcıoğlu Data Science Institute, University of California, San Diego, San Diego, CA, USA; 10 These authors contributed equally; 11 These authors contributed equally; 12 Present address: Stanley Center for Psychiatric Research, Broad Institute, Massachusetts Institute of Technology, Cambridge, MA 02142, USA; 13 Present address: Precision Biomarker Labs, Cedars-Sinai, 8700 Beverly Blvd., Davis 2904, Los Angeles, CA 90048, USA; 14 Present address: School of Life Sciences, Arizona State University, Tempe, AZ 85281, USA; 15 Present address: Department of Biology, College of Science and Mathematics, Fresno, CA 93740, USA; 16 Lead contact

## Abstract

The gastrointestinal (GI) tract is innervated by intrinsic neurons of the enteric nervous system (ENS) and extrinsic neurons of the central nervous system and peripheral ganglia. The GI tract also harbors a diverse microbiome, but interactions between the ENS and the microbiome remain poorly understood. Here, we activate choline acetyltransferase (ChAT)-expressing or tyrosine hydroxylase (TH)-expressing gut-associated neurons in mice to determine effects on intestinal microbial communities and their metabolites as well as on host physiology. The resulting multi-omics datasets support broad roles for discrete peripheral neuronal subtypes in shaping microbiome structure, including modulating bile acid profiles and fungal colonization. Physiologically, activation of either ChAT^+^ or TH^+^ neurons increases fecal output, while only ChAT^+^ activation results in increased colonic contractility and diarrhea-like fluid secretion. These findings suggest that specific subsets of peripherally activated neurons differentially regulate the gut microbiome and GI physiology in mice without involvement of signals from the brain.

## In brief

Griffiths et al. examine how activating ChAT^+^ or TH^+^ gut-associated neurons shapes the gut microbiome and physiology in mice. They uncover unique alterations in the metagenome, transcriptome, metabolome, and proteome, as well as GI motility, suggesting that subpopulations of peripherally activated neurons differentially regulate the gut microbiome and GI function with no involvement of signals from the brain.

## Graphical abstract

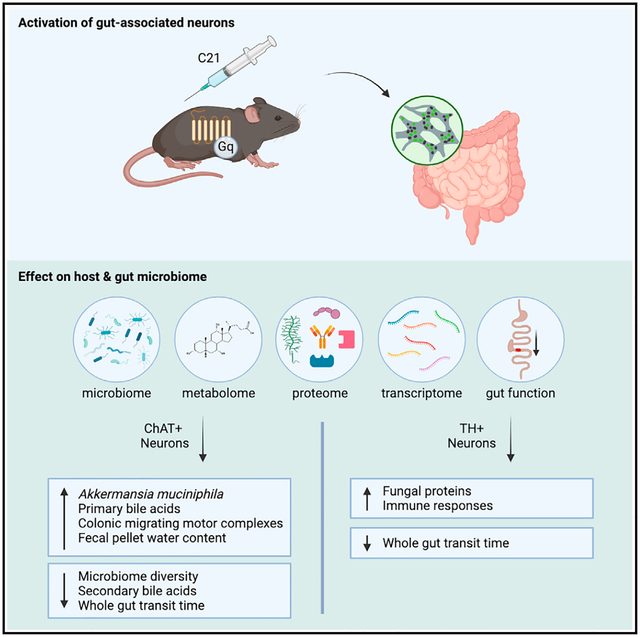

## INTRODUCTION

Diverse cell types in the gastrointestinal (GI) tract coordinate physiology within the gut^1^ and throughout the body.^2^ The mammalian gut receives and transmits neuronal signals through ~100,000 extrinsic nerve fibers originating from the sympathetic, parasym-pathetic, and sensory nervous systems.^3^ The GI tract is also innervated by an extensive network of over 100 million intrinsic neurons organized into two distinct compartments within the GI tract; namely, the myenteric plexus and submucosal plexus.^4^ The neurons of the GI tract, composing the enteric nervous system (ENS), have been implicated in processes including digestion,^5^ immunity,^6,7^ and even complex behaviors,^8^ in mice. Interactions between neurons of the GI tract and other cell types highlight the diverse roles of the ENS. For example, neuronal pathways in the gut regulate nutrient sensation through intestinal enteroendocrine cells,^9^ modulate the epithelial barrier and mucosal immunity,^10–12^ and dynamically interface with the microbiome.^13,14^ Exposure of the ENS to changing diet, microbiome, and xenobiotics creates inputs distinct from those in the central nervous system (CNS); i.e., the brain and spinal cord.

Choline acetyltransferase (ChAT) and tyrosine hydroxylase (TH) are the rate-limiting enzymes in acetylcholine and catecholamine biosynthesis, respectively, and are key chemical mediators of neurotransmission in the brain and the periphery. Acetylcholine is the primary excitatory neurotransmitter of the gut, and cholinergic neurons represent 60% of the ENS, mediating intestinal propulsion and secretion.^15,16^ Several studies have established correlations between neuronal activity, abundance, and specific physiological outcomes.^17–19^ For example, age-associated reduction of ChAT^+^ neurons in the ENS coincides with constipation and evacuation disorders,^15,16^ and clinical studies have shown that anticholinergic drugs cause constipation and cholinergic agonists can cause diarrhea.^20,21^ In a disease context, cholera toxin induces hypersecretion and sustained activation of submucosal ChAT^+^ neurons in mice.^22,23^ Although less characterized, TH^+^ neurons and dopamine signaling pathways have also been shown to affect GI motility,^24^ and TH^+^ neuronal damage in individuals with Parkinson’s disease (PD) correlates with increased constipation.^25,26^

Though known to be important for motility and secretomotor function, ChAT^+^ and TH^+^ neurons have not yet been systematically characterized and interrogated for their roles in GI physiology.^17,27^ One barrier to modulation of neuronal populations in the ENS is its size: 35–40 cm in mice. To circumvent the need for direct delivery of effectors, we leveraged a systemically delivered engineered adeno-associated virus (AAV) with enhanced tropism for the ENS and other peripheral ganglia of mice.^28^ Importantly, this vector, AAV-PHP.S, does not transduce the CNS, allowing us to uncouple peripheral activation from brain-to-gut signaling. We find that activating gut-associated ChAT^+^ and TH^+^ neurons of mice with chemogenetic modulators^29^ alters the transcriptional and proteomic landscape of the intestines as well as the gut metagenome and metabolome. Multiomics analyses allow us to characterize detailed and complex host-microbial interactions and enable prediction of neuronal influence on a number of biological processes in the gut, including providing insights into secondary bile acid production and control of fungal populations, among other interesting associations. In addition, we show that activation of gut-associated neurons strikingly impacts GI function, including motility and fluid secretion. Together, this work reveals differential effects of non-brain activation of ChAT^+^ and TH^+^ neurons in shaping the gut environment and GI physiology and generates rich datasets as a resource for further exploration (https://doi.org/10.5281/zenodo.10525220, https://github.com/mazmanianlab/Griffiths_Yoo_et_al/).

## RESULTS

### Distinct spatial localization of ChAT^+^ and TH^+^ neurons in the ENS

Broad ENS morphology has been characterized previously using immunohistochemistry (IHC).^16,30,31^ To map neurons in mice with higher resolution, we used recombinant AAVs to fluorescently label enteric neurons *in vivo* and tissue-clearing techniques to enhance visualization of intact GI tissue.^32–34^ Imaging whole tissue, without the need for sectioning, preserves neuronal architectures over large distances and across both longitudinal and cross-sectional axes. The AAV capsid variant AAV-PHP.S is optimized for systemic delivery in mice^35^ and displays increased tropism for the peripheral nervous system (PNS), including the ENS.^28^ To further optimize ENS expression, we replaced the CAG promoter used previously^28^ with the human synapsin 1 (hSYN1) promoter, which has been shown to restrict gene expression to neurons^28^ and minimize expression in peripheral targets, such as the dorsal root ganglia (DRGs).^36^ To assess off-target effects, we compared expression of AAV-PHP.S-delivered hSYN1-mNeonGreen to that of CAG-mNeonGreen in various non-ENS tissues known to affect GI function (Figure S1). Expression from the hSYN1 construct only sparsely labeled the DRGs and jugular-nodose ganglia and did not label neuronal projections in the vagus nerve or dorsal root, unlike the previously used CAG construct (Figures S2A and S2B).^28^ In the CNS, AAV-PHP.S-hSYN1 did not label neurons in the brain, brain stem, or spinal cord (Figure S3).

**Figure 1.**
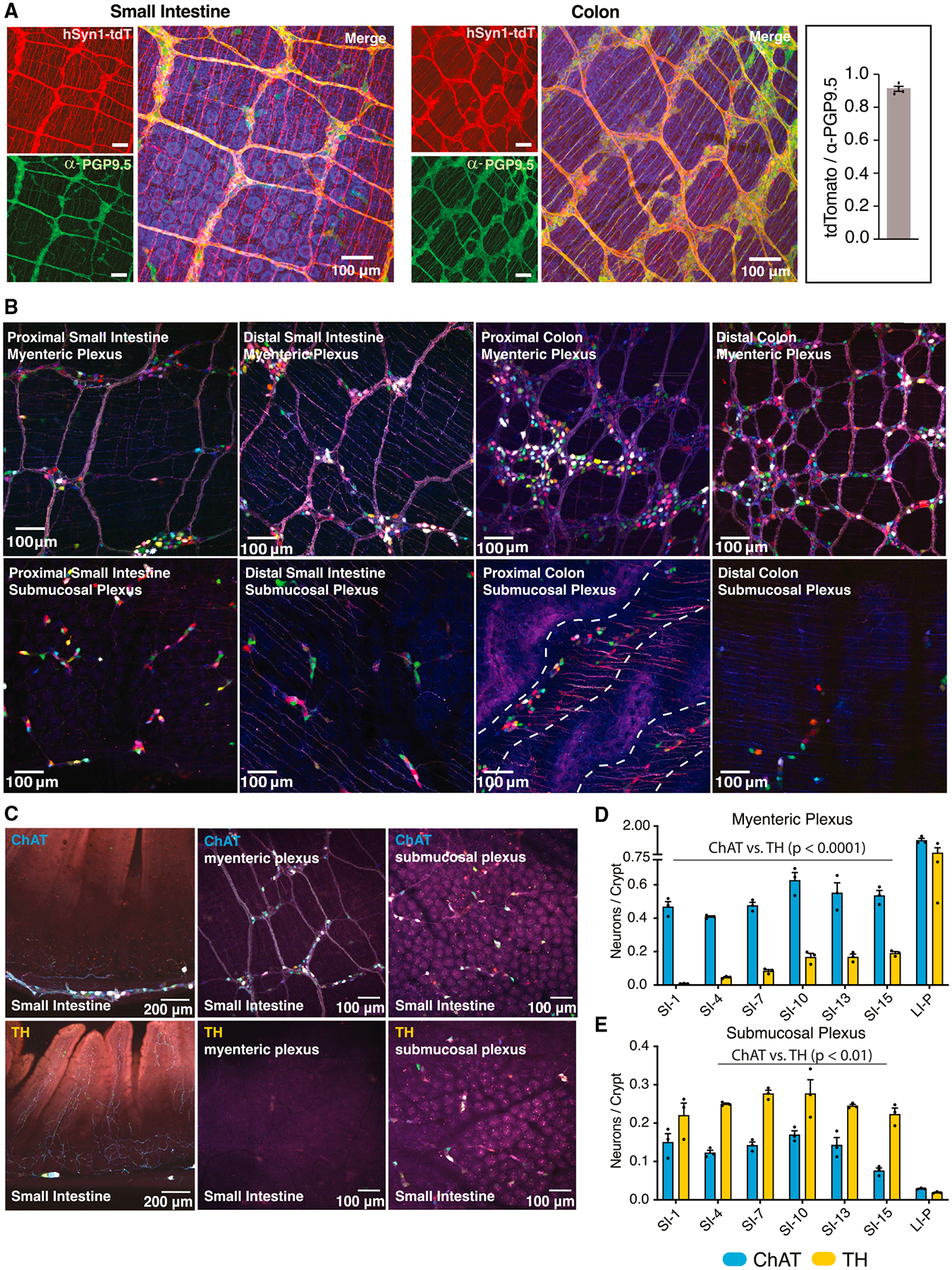
ChAT^+^ versus TH^+^ neuronal distribution in the ENS (A) Representative images of SI and colon from mice infected with AAV-PHP.S-hSYN1-tdTomato and immunolabeled with the pan-neuronal antibody PGP9.5. Inset: quantification of the ratio of tdTomato^+^ cells/PGP9.5^+^ cells (n = 3 mice; each data point represents the average of 3 representative fields). (B) Representative images of proximal and distal regions of the SI and colon from AAV-PHP.S-hSYN1-XFP-infected mice. Dotted lines demarcate the rugae (folds) in the proximal colon. (C) Representative images of cross-sections and myenteric and submucosal plexuses in ChAT-Cre and TH-Cre mice infected with AAV-PHP.S-hSYN1-DIO-XFP. (D and E) Density of neurons in the myenteric plexus and submucosal plexus of ChAT-Cre and TH-Cre mice normalized to the number of crypts (n = 3 mice; each data point represents the average of 3 representative fields). ChAT-Cre vs. TH-Cre mice were compared using two-way ANOVA with Sidak’s correction for multiple comparisons for the SI and LI independently. Comparison of different regions in the SI of ChAT-Cre or TH-Cre mice was analyzed using one-way ANOVAs with Tukey’s correction for multiple comparisons. See also Figures S1–S5 and Video S1. Source data can be found at https://github.com/mazmanianlab/Griffiths_Yoo_et_al/tree/main/ENS%20quantification.

We packaged genes encoding fluorescent proteins (tdTomato or mNeonGreen) under control of the hSYN1 promoter into AAV-PHP.S, delivered them systemically, and found that 90% (±2.6% SD) of ENS cells labeled with antibodies against protein gene product 9.5 (PGP9.5), a pan-neuronal protein, co-localized with virally labeled neurons in the small intestine (SI) and colon (Figure 1A). A single systemic injection of AAV-PHP.S-hSYN1-mNeonGreen at a dose of 10^12^ viral genomes (vg) was sufficient to label spatially diverse regions of the ENS, such as ganglia proximal and distal to the mesentery (Figure S4A). Viral transduction was uniform throughout the SI and colon, aside from a small (~1.5-cm) section of the medial colon that, for unknown reasons, was consistently not well transduced and was therefore excluded from further analysis (Figure S4B).

To explore the general architecture of the ENS, we transduced wild-type mice with a single intravenous (i.v.) injection of a pool of AAV-PHP.S packaging multiple fluorescent proteins (AAV-PHP.S-hSYN1-XFP), which broadly labeled enteric neurons in the gut and enabled us to distinguish cells by distinct colors resulting from stochastic transduction with different combinations of XFPs (Figure 1B). We quantified the number of neurons and ganglia as well as the ganglion size (i.e., the number of neurons in each ganglion) in the myenteric and submucosal plexuses of seven regions of the SI and two regions of the colon (Figures S5A–S5F). Regions were approximately 1 cm in length, and the tissue was sampled every 2–3 cm. We saw that, in the SI, the numbers of neurons and ganglia generally increased toward the distal portion of the myenteric plexus, while the converse was true for the submucosal plexus (i.e., lower numbers in distal than proximal regions) (Figures S5A and S5B). Additionally, the size of the ganglia (i.e., the number of neurons per ganglion) increased in the distal region of the SI myenteric plexus, a feature not observed in the submucosal plexus (Figure S5C). While neuronal numbers were similar in the proximal and distal regions of the colonic plexuses (Figure S5D), the number of myenteric ganglia increased (Figure S5E) while the size of each ganglion decreased in the distal colon (Figure S5F). Interestingly, submucosal neurons in the proximal colon localized to natural folds in the tissue (Figure 1B, dashed lines, bottom, second from right).

To visualize ChAT^+^ and TH^+^ neurons, we employed mouse lines in which Cre recombinase (Cre) is expressed under the control of the respective gene promoter and engineered viral constructs with the transgene in a double-floxed inverted orientation (DIO) so that the transgene is flipped and expressed in a Cre-dependent manner. After transducing ChAT-Cre or TH-Cre mice with AAV-PHP.S-hSYN1-DIO-XFP, we observed that both neuronal populations occupy spatially distinct layers of the GI tract, with ChAT^+^ neurons primarily located in the myenteric plexus and TH^+^ neurons more abundant in the submucosal plexus (Figure 1C). Quantifying this effect, we found more ChAT^+^ than TH^+^ neurons in all assayed regions of the myenteric plexus (Figure 1D), although the density of TH^+^ myenteric neurons increased distally (Figure 1D; 10-fold increase from SI-1 vs. SI-7; 2-fold increase from SI-7 vs. SI-10/13/15). In the SI, by contrast, there were more TH^+^ than ChAT^+^ submucosal neurons (Figure 1E). In addition to providing these insights into ENS architecture, this approach for whole-tissue imaging without the need for antibody labeling (which has limited penetration to deeper layers) should be broadly useful for profiling other neuronal and non-neuronal cell types in the gut.

### Activation of gut-associated neurons reshapes the gut microbiome

The unique spatial organization of ChAT^+^ and TH^+^ neurons we observed suggests potentially distinct functions, which we decided to investigate through specific activation of each neuronal population. First, we examined the specificity of AAV-PHP.S-hSYN1 by staining gut-extrinsic PNS ganglia for TH and found no transduction of TH^+^ cells in the DRGs or jugular-nodose ganglia (Figures S2B and S2C). ChAT^+^ neurons are absent in these peripheral ganglia (Figure S2C).^37,38^ Prior research has shown that AAV-PHP.S-hSYN1 transduces the prevertebral sympathetic ganglia, which are known to innervate the gut,^39^ but these ganglia also lack ChAT^+^ neurons.^40^ In fact, the vast majority of neurons in the prevertebral sympathetic ganglia are TH^+^.^40,41^ Therefore, for the remainder of the manuscript, we will use the term “gut-associated” to refer to ChAT^+^ neurons in the ENS or TH^+^ neurons in the ENS plus innervating prevertebral sympathetic ganglia.

For cell-specific neuronal activation, we employed a Cre-dependent genetic construct encoding an activating designer receptor exclusively activated by designer drugs (DREADD), named hM3Dq, which is a modified neurotransmitter receptor designed to induce neuronal activation when exposed to compound 21 (C21), a “designer drug” specific to this receptor.^42^ We validated functional gene delivery and expression using intestinal explants from a ChAT-Cre mouse transduced with the activating DREADD and a construct encoding the calcium sensor GCaMP6f, observing a gradual increase in fluorescence consistent with a calcium transient following administration of C21 (Figure S5G; Video S1).

**Figure 2.**
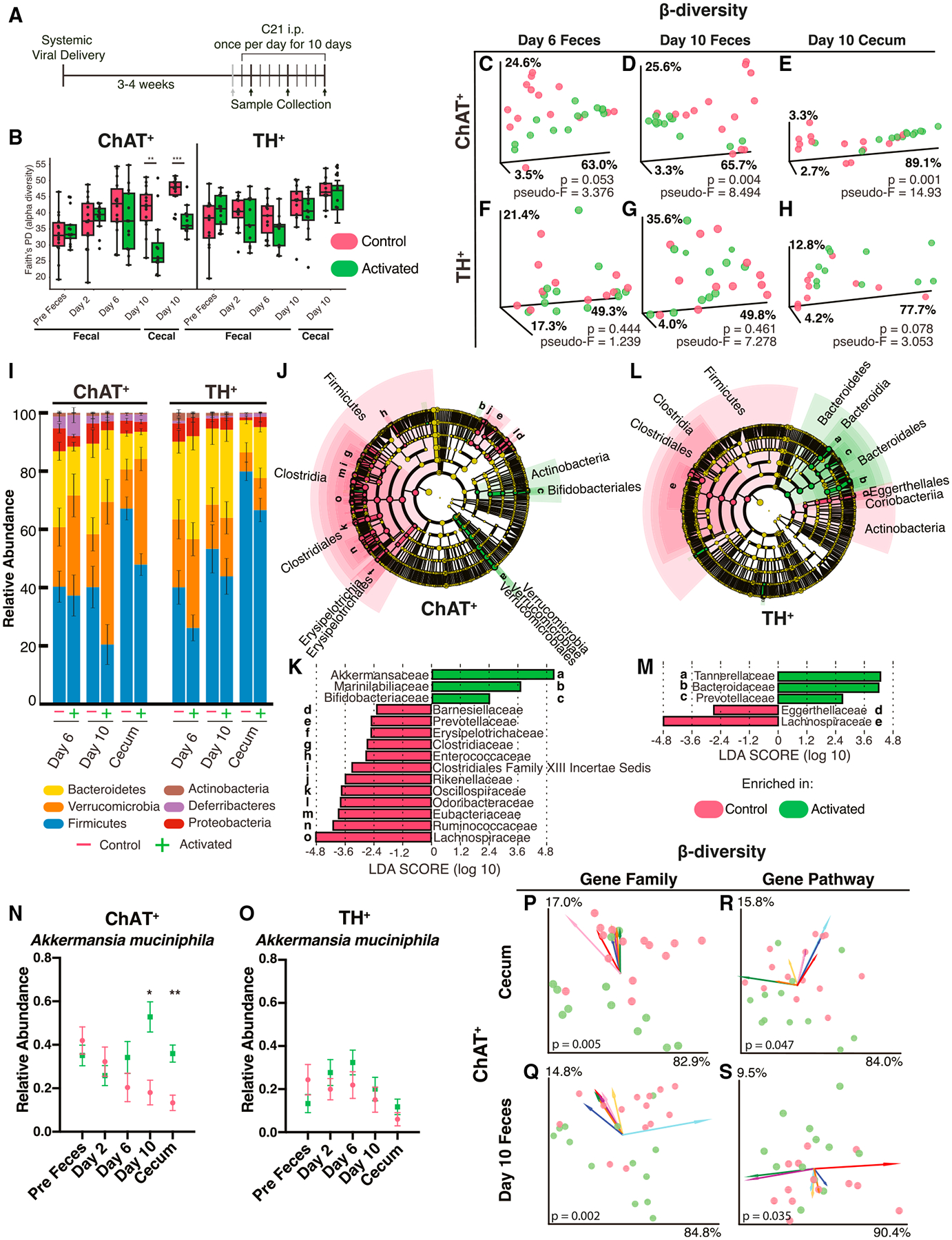
Gut-associated ChAT^+^ and TH^+^ neuronal activation alters the gut microbiome (A) Experimental paradigm. Cre-dependent hM3Dq was virally administered to either ChAT-Cre or TH-Cre mice. After 3–4 weeks of expression, C21 was injected daily for 10 days to induce specific neuronal activation. Feces was sampled the day prior to the first C21 injection and on days 2, 6, and 10 of C21 administration, and tissue and cecal contents were collected 1 h after the last injection. (B) Faith’s phylogenetic diversity of feces and cecal contents over 10 days of neuronal activation in ChAT^+^ and TH^+^ mice. Feces was collected pre-experiment (1 day before the first injection) and on days 2, 6, and 10. Cecal contents were collected at the experimental endpoint on day 10. **p < 0.01, ***p < 0.001, determined by Kruskal-Wallis one-way ANOVA. (C–H) Weighted UniFrac principal-coordinate analysis (PCoA) of activated vs. control in ChAT^+^ and TH^+^ mice. Statistics were performed in QIIME2 as in Bolyen et al.^44^ (I) Stacked bar graph showing phylum-level changes in relative abundance on day 6 and day 10 of injection for feces and day 10 for cecal contents. (J–M) Linear discriminant analysis effect size (LEfSe) of the cecal microbiome. Cladograms show differential phylogenetic clusters and family-level differences in activated and control (J and K) ChAT^+^ and (L and M) TH^+^ mice. Cutoff: log_10_(LDA score) > 2 or < −2. (N and O) Changes in relative abundance of *A. muciniphila* in feces and cecal contents of (N) ChAT^+^ and (O) TH^+^ mice (n = 11–14 mice per group per time point). Red, control; green, hM3Dq-activated. *p < 0.05, **p < 0.01, determined by multiple t tests with Holm-Sidak correction for multiple comparisons. (P–S) Beta diversity of bacterial gene families and pathways in the (P and R) cecum and (Q and S) post-9 feces of control and activated mice. The direction and length of arrows indicate their influence in separating control and activated groups. Colors represent gene families and pathways (annotated in Figure S7). See also Figures S6 and S7. Source can be found at https://github.com/mazmanianlab/Griffiths_Yoo_et_al/tree/main/metagenomics.

We reasoned that neuronal activation in the gut may impact the composition and community structure of the gut microbiome. Accordingly, we transduced ChAT-Cre or TH-Cre mice with either virus carrying the activating DREADD (AAV-PHP.S-hSYN1-DIO-hM3Dq-mRuby2) or a control virus expressing only the fluorescent reporter protein (AAV-PHP.S-hSYN1-DIO-mRuby2). We performed shotgun metagenomics on a longitudinal series of fecal samples collected prior to and following ChAT^+^ or TH^+^ neuron activation by C21 (on days 2, 6, and 10 of C21 administration) as well as contents of the terminal cecum collected on day 10 (Figure 2A). In ChAT^+^-activated mice, Faith’s phylogenetic diversity (i.e., alpha diversity) decreased dramatically in the day 10 fecal and cecal samples (Figure 2B), with many microbial taxa less abundant (Figures 2I–2K, S6, and S7). In contrast, TH^+^-activated mice displayed phylogenetic diversity similar to controls throughout the experiment (Figure 2B). Using weighted UniFrac distances and principal-coordinate analysis (PCoA) to determine the composition of microbial communities (i.e., beta diversity), we observed a distinction between ChAT^+^-activated and control animals in both feces and cecal contents, a shift that was absent in samples from TH^+^-activated mice and controls (Figures 2C–2H). Over the experimental time course, Verrucomicrobia became significantly enriched in ChAT^+^-activated mice (Figure 2I). To explore differentially abundant bacterial taxa, we used linear discriminant analysis effect size (LEfSe)^43^ and generated cladograms depicting the phylogenetic relationships of differentially abundant taxa (Figures 2J–2M). This analysis revealed that the bacterial species *Akkermansia muciniphila* drove the increase in Verrucomicrobia we observed in ChAT^+^-activated mice (Figures 2N and 2O).

In addition to identifying microbial species, metagenomic analysis can reveal gene families and pathways that are differentially abundant in the microbiome. ChAT^+^-activated mice, but not TH^+^-activated mice, showed changes in beta diversity of both gene families and pathways, with shifts evident in the cecal contents and feces collected 9 days after activation (Figures 2P–2S). The most distinguishing features were highly represented in the control group and downregulated in ChAT^+^-activated mice and were mainly associated with bacterial processes, such as nucleotide biosynthesis and metabolism and protein translation and transport (Figures 2P–2S, S7C, and S7D). This downregulation is consistent with the decrease in bacterial alpha diversity we observed in ChAT^+^-activated mice (Figure 2B). We conclude that neuronal activation actively reshapes the gut microbiome at community, species, and genetic levels, with considerable differences between the effects of ChAT^+^ and TH^+^ neurons.

### Neuronal stimulation impacts the gut metabolome

**Figure 3.**
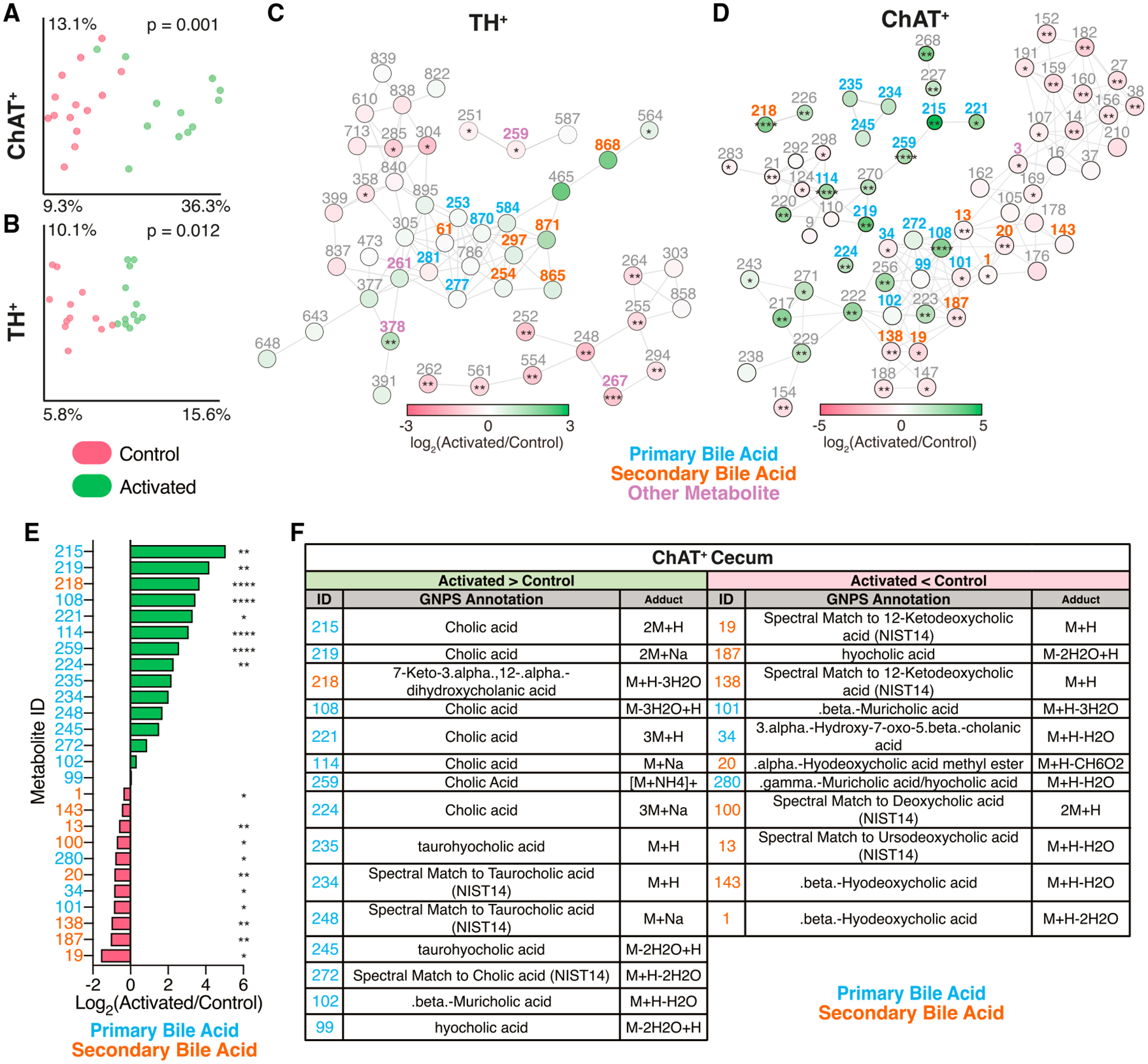
Gut-associated ChAT^+^ or TH^+^ neuronal activation alters host and microbe-derived luminal metabolites (A and B) Canberra PCoA of the cell-free, luminal metabolome of cecal contents from control (red) and activated (green) ChAT^+^ and TH^+^ mice. Statistical analyses were performed in QIIME2 as in Bolyen et al.^44^ (C and D) Metabolic networks constructed from identified cecal metabolites in TH^+^ and ChAT^+^ mice. Each node is colored by its upregulation (green) or downregulation (red) in the activated group and is labeled with an ID number corresponding to annotation, mass-to-charge ratio, retention time, fold change, and significance value in Table S1. (E) Fold changes of specific bile acids identified as upregulated (green bars) or downregulated (red bars) in activated ChAT^+^ mice. (F) Annotations of bile acids highlighted in (E). Metabolite IDs are colored according to annotation as primary (blue) or secondary (orange) bile acids. Metabolite IDs are specific to each sample (n = 12–14 for each group analyzed). *p < 0.05, **p < 0.01, ***p < 0.001, ****p < 0.0001. See also Table S1.

Given the intimate and intertwined mouse and microbial co-metabolism, the changes in the microbial metagenome we observed in response to neuronal activation led us to predict that there would also be alterations in the profile of gut metabolites. We therefore performed untargeted metabolomics using liquid chromatography-tandem mass spectrometry (LC-MS/MS) to assay molecular changes in cecal contents and feces following neuronal activation in the gut. In both ChAT^+^- and TH^+^-activated neurons, compared with unactivated controls (no DREADD), we observed a strong separation of metabolome profiles in cecal samples taken 1 h following the last C21 injection (Figures 3A and 3B). Thus, targeted activation of ChAT^+^ and TH^+^ gut-associated neurons appears to strongly influence the gut metabolome.

To contextualize these data, we applied the Global Natural Products Social Molecular Networking (GNPS) tool,^45^ an openaccess MS repository and analysis pipeline. GNPS revealed metabolic networks of both annotated and unannotated molecules in the cecal contents of ChAT^+^-activated and TH^+^-activated mice (Figures 3C and 3D), allowing us to identify metabolites with differential abundance between control and activated samples. Activation of TH^+^ neurons strongly increased metabolites whose closest spectral matches were linoelaidic acid (ID: 626), oleanolic acid methyl ester (ID: 378), and coproporphyrin I (ID: 739). Metabolites that spectrally resembled xanthine (ID: 259), genistein (ID: 846), and *trans*-ferulic acid (ID: 707) were decreased upon activation of TH^+^ neurons (Table S1).

In both ChAT^+^-activated and TH^+^-activated mice, the molecular networks largely consisted of level 3 annotations (based on the Metabolomics Standards Initiative [MSI]^46^) of compounds belonging to the bile acid molecular family and their conjugates as well as unannotated analogs (Figures 3C and 3D). Primary bile acids are chemicals derived from host (mouse) cholesterol biosynthesis, which are subsequently co-metabolized by gut bacteria into secondary bile acids.^47,48^ Interestingly, metabolites with a closest spectral match to the primary bile acid cholic acid (IDs: 108, 114, 215, 219, 221, 224, and 259) were significantly enriched in the cecum of ChAT^+^-activated mice (Figures 3D–3F; Table S1). Additional metabolites that spectrally resemble tauro-conjugated primary bile acids, such as taurocholic acid (IDs: 234 and 248) and taurohyocholic acid (ID: 235), trended upwards. Conversely, features matching the spectra of secondary bile acids and bile acid metabolites, such as ursodeoxycholic acid (ID: 13), deoxycholic acid (ID: 100), beta-hyodeoxycholic acid (IDs: 1 and 143) and 12-ketodeoxycholic acid (IDs: 19 and 138) were decreased in ChAT^+^-activated mice (Figures 3D–3F). These data suggest that activation of ChAT^+^ neurons may modulate, either directly or indirectly, primary bile acid secretion and/or metabolism to secondary bile acids, which have been implicated in a number of metabolic and immunologic functions, as discussed below.

### Neuronal subpopulations differentially shape the gut luminal proteome

**Figure 4.**
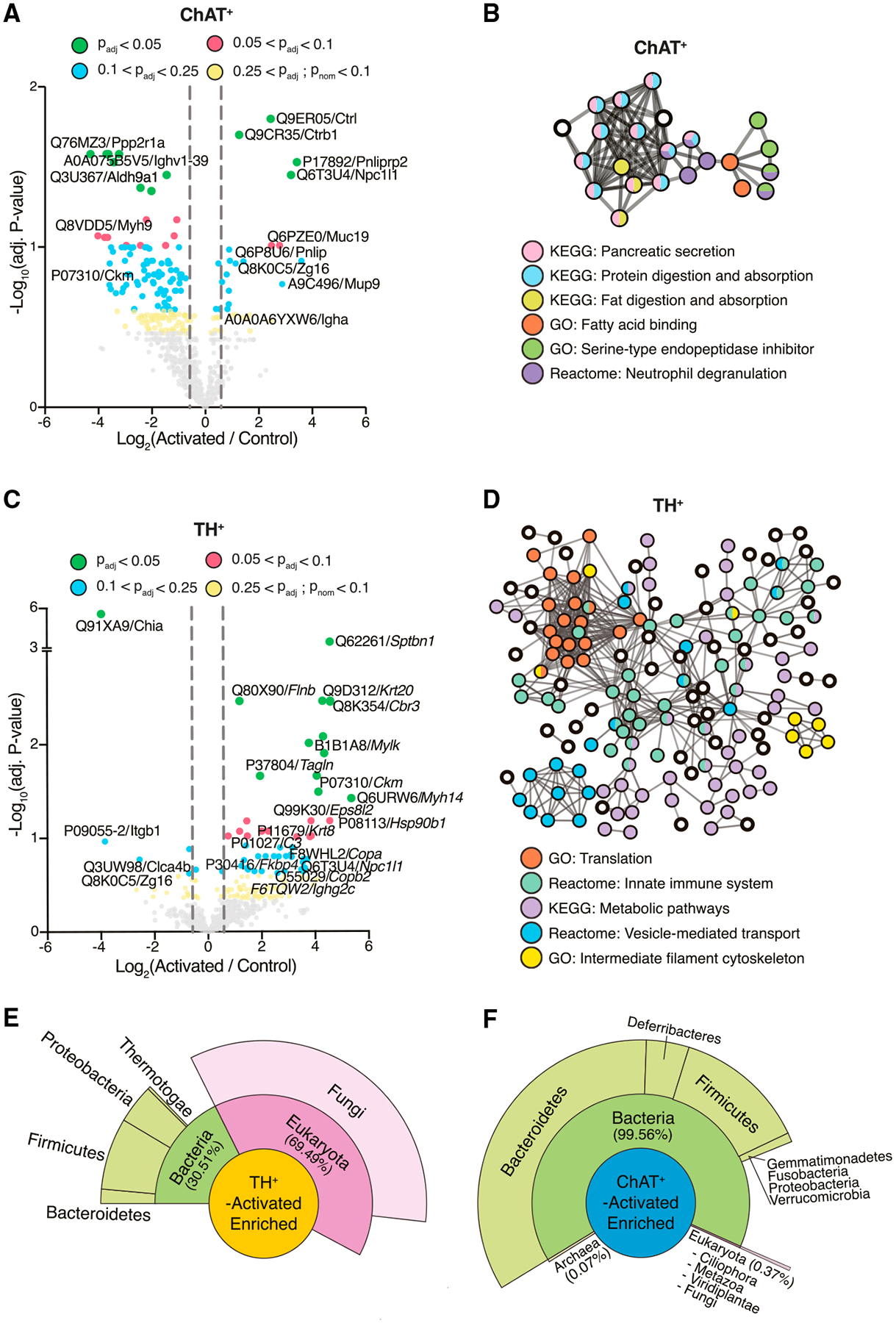
Gut-associated ChAT^+^ or TH^+^ neuronal activation alters host and microbe-derived luminal proteins (A) Volcano plot of differentially expressed host proteins identified in the cecal contents of ChAT^+^-activated (n = 8) vs. ChAT^+^ control (n = 9) mice 1 h after final C21 administration. (B) STRING network analysis of host proteins that were more abundant in ChAT^+^-activated mice (p_nom._ < 0.2). (C) Proteomics volcano plot of TH^+^-activated vs. TH^+^ control mice (n = 7 mice per group). (D) STRING network analysis of upregulated host proteins in TH^+^-activated mice (p_nom._ < 0.2). (E and F) Unipept metaproteomics analysis of upregulated microbial proteins (fold change > 2, p_nom._ < 0.2) in TH^+^-activated and ChAT^+^-activated mice (n = 7–9 mice per group). Source data for (A) can be found at https://github.com/mazmanianlab/Griffiths_Yoo_et_al/blob/main/proteomics/CHAT_proteomics_volcano.txt. Source data for (C) can be found at https://github.com/mazmanianlab/Griffiths_Yoo_et_al/blob/main/proteomics/TH_proteomics_volcano.txt. Source data for (E) and (F) can be found at https://github.com/mazmanianlab/Griffiths_Yoo_et_al/blob/main/proteomics/metaproteomics/Microbiome_associated_proteins.xlsx.

Proteins from the mouse, gut microbes, and diet converge and interact in the GI tract.^49^ We performed untargeted label-free proteomics by LC-MS/MS of cell-free supernatants of the cecal contents from ChAT^+^-activated and TH^+^-activated mice and controls collected 1 h following the final C21 treatment (Figure 2A). Consistent with the increase in cecal bile acid metabolites that we observed in ChAT^+^-activated mice, we report an increased abundance of Niemann-Pick C1-like 1 protein (NPC1L1) in the cecum of these mice (Figure 4A). NPC1L1 is expressed on the apical surface of enterocytes and is integral to the absorption of free cholesterol, the precursor of bile acids, from the lumen.^50^ Goblet cell-related proteins, specifically Mucin-19 (MUC19) and Zymogen granule 16 (ZG16), a protein localized to secretory granules,^51^ also trended upward following ChAT^+^ neuronal activation (Figure 4A). Conversely, one of the most highly downregulated proteins was an aldehyde dehydrogenase (Q3U367) encoded by the *Aldh9a1* gene, which is involved in the catalytic conversion of putrescine to γ-aminobutyric acid (GABA).^52^ While GABA is the primary inhibitory neurotransmitter in the CNS, little is known about its role in the ENS. The most significantly upregulated proteins in cecal contents of ChAT^+^-activated mice were pancreatic digestive enzymes, including chymopasin (CTRL), chymotrypsinogen B1 (CTRB1), and pancreatic lipase-related protein 2 (PNLIPRP2) (Figure 4A). Accordingly, network analysis of upregulated proteins revealed that KEGG pathways associated with digestion represent the majority of the network (Figure 4B). This is consistent with evidence that cholinergic, viscerofugal neurons send signals from the GI tract to other organs of the digestive system, including the pancreas.^4^ Cholinergic innervation of the pancreas plays a significant role in regulating pancreatic functions, such as the secretion of digestive enzymes and insulin release.^53^

Peripheral activation of TH^+^ gut-associated neurons also altered the luminal proteome of the cecum. Notably, 88% (52 of 59) of the differentially abundant proteins (p_adj._ < 0.25) were distinct from those identified in ChAT^+^-activated mice. The overall direction of the effect was also reversed: ~90% of differentially abundant cecal proteins in TH^+^-activated mice were upregulated (53 of 59) compared with ~18% in ChAT^+^-activated mice (20 of 112), suggesting that activation of distinct neuronal subsets is associated with opposing changes in GI function. We observed signatures of increased protein-protein interactions in cecal contents of TH^+^-activated mice, evidenced by more network nodes and connections (Figure 4D). Filamin B (FLNB) and spectrin β chain, non-erythrocytic 1 (SPTBN1) were two of the most significantly enriched proteins following TH^+^ neuron activation (Figure 4C). Both are associated with the intestinal brush border and membrane vesicles.^54,55^ Accordingly, coatomer proteins also trended upward (COPA and COPB2) (Figure 4C), and vesicle-mediated transport was one of the major protein networks altered (Figure 4D). Other upregulated protein interaction networks were associated with metabolic pathways, ribosomal activity, and the immune system (Figure 4D). For example, the immune-related proteins immunoglobulin heavy constant alpha (IGHA) (in ChAT^+^-activated), immunoglobulin heavy constant gamma 2C (IGHG2C), and complement component 3 (in TH^+^-activated) trended upward (Figures 4A and 4C).

Perhaps the most intriguing observation was the strong depletion of acidic mammalian chitinase (CHIA) upon activation of TH^+^ neurons (Figure 4C). Chitin is a natural polysaccharide that is a major component of fungal cell walls,^56^ but intestinal chitinases are poorly studied in mice. This result prompted us to query the pan-proteomics dataset against a microbial protein database, which revealed that the decrease in CHIA abundance following TH^+^ neuron activation was accompanied by a large bloom in fungus-associated peptides in the microbiome (~59% of peptides mapped to any microbe) (Figure 4E). In contrast, fungal peptides represented only ~0.4% of enriched peptides in the lumen of ChAT^+^-activated mice (Figure 4F). Unfortunately, we were unable to corroborate these proteomics data with metagenomics since the DNA extraction method we used was not optimized for fungi. However, these findings suggest that the reduced chitinase production of activated TH^+^ cells is directly associated with a dramatic increase in fungal proteins, which, if experimentally validated in the future, would represent a circuit by which the gut-associated neurons of mice regulate fungal load in the gut.

### Activation of ChAT^+^ and TH^+^ neurons alters the intestinal transcriptome

**Figure 5.**
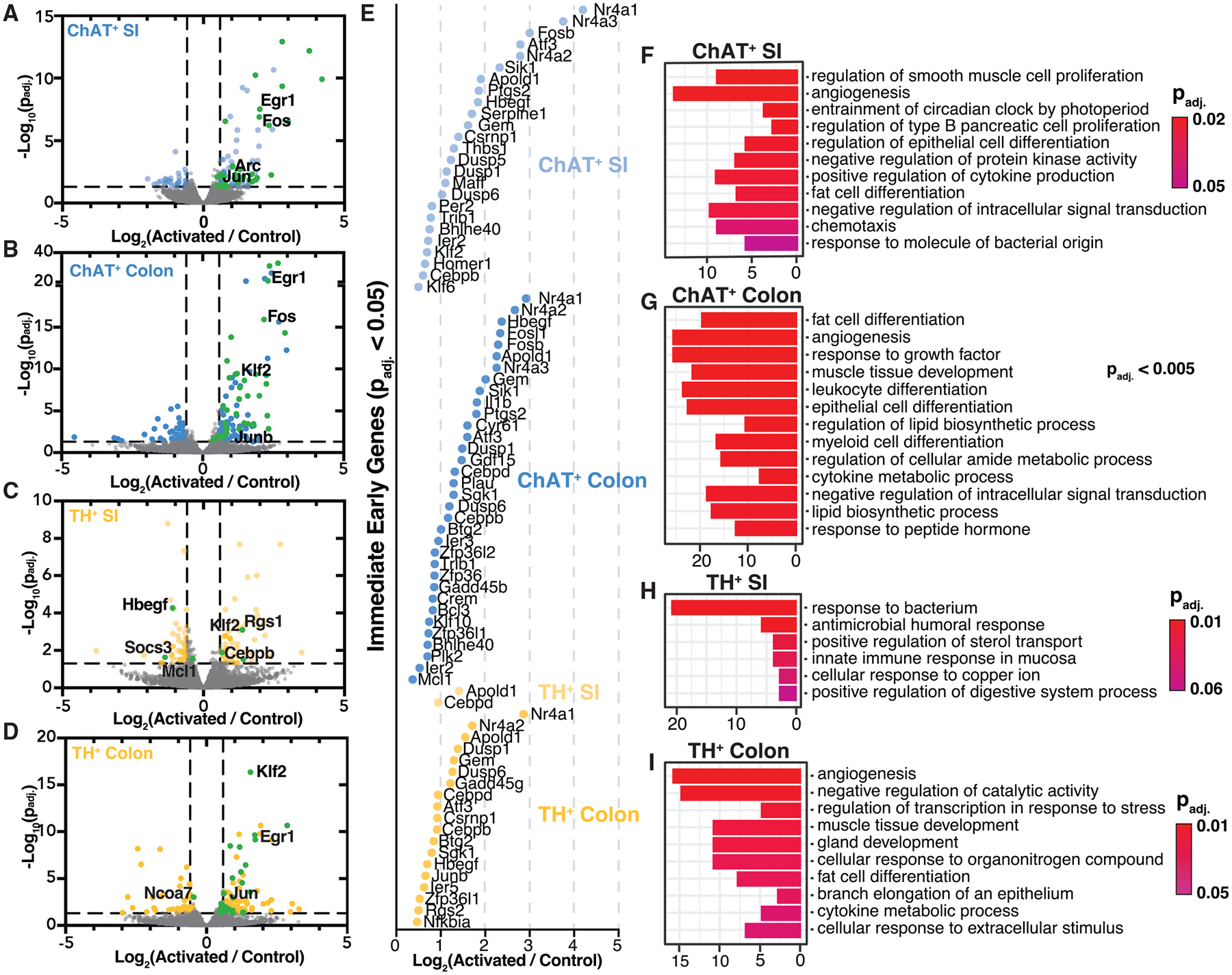
ChAT ^+^ and TH^+^ activation-mediated transcriptomic changes (A–D) DEGs in DREADD-activated vs. control (A) ChAT ^+^ distal SI, (B) ChAT^+^ proximal colon, (C) TH^+^ distal SI, and (D) TH^+^ proximal colon. Dashed vertical lines, fold change (FC) ± 1.5; dashed horizontal lines, p_adj._ < 0.05. Transcripts of IEGs are highlighted in green and annotated. (n = 10 mice per group). (E) FC of upregulated IEGs (p_adj._ < 0.05) as defined previously.^57^ (F–I) GSEA of Gene Ontology (GO) terms for (F) ChAT^+^ distal SI, (G) ChAT^+^ proximal colon, (H) TH^+^ distal SI, and (I) TH^+^ proximal colon. See also Tables S2 and S3. Source Can be found at https://github.com/mazmanianlab/Griffiths_Yoo_et_al/tree/main/RNAseq.

Given the changes to the gut microbiome, proteome, and metabolome that we observed, we were interested in the tissuelevel impact of neuronal activation on the intestinal transcriptome. We therefore profiled gene expression with QuantSeq, a quantitative 3′ mRNA sequencing technology, in 1 cm of tissue from the distal SI and proximal colon harvested 1 h after the last C21 injection. Rapid and transient expression of immediate-early genes (IEGs) is widely used as a measure of increased neuronal activity,^57^ and the IEGs *Fos*, *Egr1*, *Jun*, and *Klf2* were among the most significantly upregulated transcripts we identified in the SI and colon of both ChAT^+^- and TH^+^-activated mice (Figures 5A–5D). These IEGs are also known to be upregulated during growth and differentiation of highly active cell types, such as immune cells,^58,59^ smooth muscle cells,^60^ and intestinal epithelial cells.^61^

In the distal SI, we found similar numbers of differentially expressed genes (DEGs; p_adj._ < 0.05) in ChAT^+^-activated mice (162 DEGs) and TH^+^-activated mice (165 DEGs) (Figures 5A and 5C). The direction of regulation differed, however, with ~73% of DEGs upregulated upon ChAT^+^ activation (118 upregulated and 44 downregulated) and ~58% of DEGs downregulated upon TH^+^ activation (69 upregulated and 96 downregulated). IEGs followed this overall pattern, with 29 upregulated in the distal SI of ChAT^+^-activated mice but only two upregulated in TH^+^-activated mice (Figure 5E) and three (i.e., *Hbegf*, *Soca3*, and *Mcl1*) downregulated (Figure 5C). Similar proportions of DEGs were upregulated in the proximal colon of both ChAT^+^-activated (169 upregulated and 84 downregulated) and TH^+^-activated mice (130 upregulated and 62 downregulated) (Figures 5B and 5D). Given the enrichment in fungal proteins and reduction in the level of the CHIA protein in the TH^+^-activated mice, we explored potential immune responses to fungi but found no obvious inflammatory signals compared with control mice (Table S3).

To gain insight into the cellular functions of DEGs, we used gene set enrichment analysis (GSEA) (Figures 5F–5I; Table S2). Notably, the most highly enriched gene ontology (GO) term for the distal SI of ChAT^+^-activated mice was “regulation of smooth muscle cell proliferation” (Figure 5F), whereas in TH^+^-activated mice, it was “response to bacteria” (Figure 5H), consistent with the increase in immune-related responses suggested by our proteomics dataset. In the proximal colon, we observed similar GO pathways in ChAT^+^-activated and TH^+^-activated mice (Figures 5G and 5I), suggesting that transcriptomic signatures may depend on the context of the activated neurons. In the SI, ChAT^+^ neurons predominantly border muscle cells in the myenteric plexus, while TH^+^ neurons neighbor epithelial and immune cells, which respond to bacteria in the submucosal plexus of the distal SI (Figures 1C–1E). In the colon, both neuronal subsets are abundant in the myenteric plexus (Figure 1D). In both myenteric and submucosal plexuses, we saw a wider breadth of pathways upregulated by activation of ChAT^+^ neurons than TH^+^ neurons, with ChAT^+^ neuronal activation impacting diverse cellular functions in the GI tract, involving endothelial, epithelial, immune, and adipose cells (Figure 5G; Table S2).

### Differential functional GI outcomes of activation of ChAT^+^ and TH^+^ neurons

**Figure 6.**
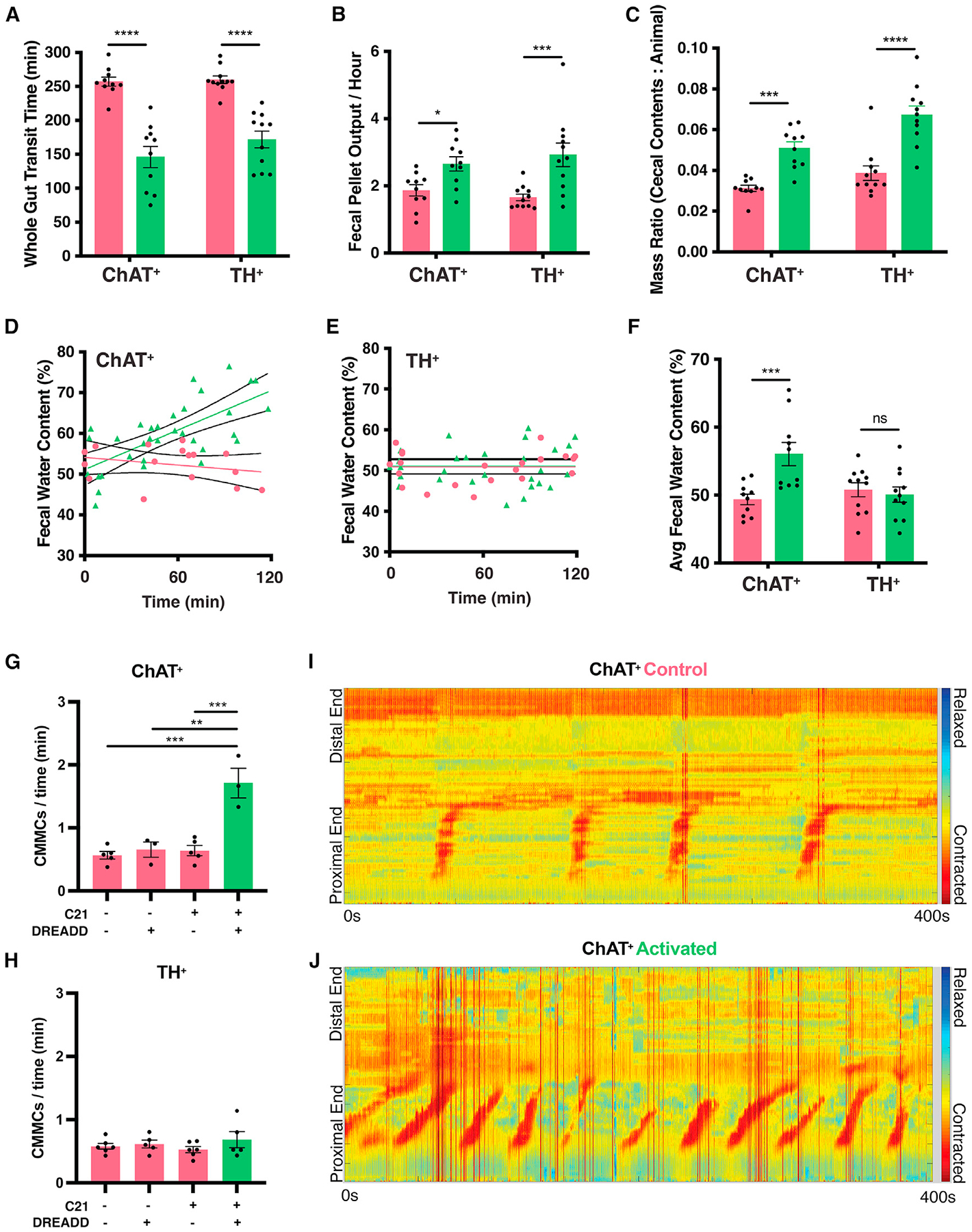
GI physiology differences in ChAT^+^ vs. TH^+^ mice following activation (A) Activation-mediated changes in whole-gut transit time in ChAT^+^ and TH^+^ mice. (B) Activation-mediated changes in fecal pellet output in ChAT^+^ and TH^+^ mice. (C) Activation-mediated changes in normalized cecal content mass in ChAT^+^ and TH^+^ mice. (D and E) Fecal pellet water content in (D) ChAT^+^ and (E) TH^+^ mice over 2 h following C21 activation, with a least-squares nonlinear regression displaying a 95% confidence interval. (F) Average fecal pellet water content in ChAT^+^ and TH^+^ mice following activation. (A–F) (n = 10–11 mice per group). *p < 0.05, ***p < 0.001, ****p < 0.0001, determined by 2-way ANOVA with Sidak′s method for multiple comparisons. (G and H) Frequency of *ex vivo* CMMCs from (G) ChAT^+^ and (H) TH^+^ mice over 30 min following activation (n = 3–6 mice per group). **p < 0.01, ***p < 0.001, determined by 2-way ANOVA with Sidak′s method for multiple comparisons. (I and J) Heatmaps showing frequency of CMMCs over 400 s following activation in *ex vivo* preparations from (I) ChAT^+^ control and (J) ChAT^+^ DREADD-administered mice. See also Figure S6 and Table S4.

Motivated by the complexity of responses we observed following activation of neuronal populations in the gut, we decided to assay functional GI outcomes. Both ChAT^+^ and TH^+^ neuronal populations are known to be important for motility and secretory function,^17,27^ but they have never been specifically modulated to study GI physiology in a freely behaving mammal. Activation of either ChAT^+^ or TH^+^ gut-associated neurons resulted in faster whole-gut transit time, increased fecal pellet output, and mass of cecal contents compared with control mice (Figures 6A–6C). Fecal pellets from ChAT^+^-activated but not TH^+^-activated mice had increased water content, which is consistent with reports in the literature of involvement of ChAT^+^ enteric neurons in fluid secretion (Figures 6D–6F).^15,21,22^ This distinction is particularly notable given the higher concentration of TH^+^ neurons than ChAT^+^ neurons in most regions of the submucosal plexus (Figure 1), which is largely responsible for fluid secretion and absorption.^1^ Daily administration of C21 for 9 days to control mice (no DREADD) did not cause any obvious health impairment, and the mice maintained body weight throughout the experimental period (Figures S6C and S6D). TH^+^-activated mice also maintained body weight, but ChAT^+^-activated animals experienced slight weight loss that likely reflects the diarrhea-like phenotype over 9 consecutive days. To further examine gut motility in the absence of extrinsic innervation, we analyzed propulsive colonic migrating motor complexes (CMMCs) in an *ex vivo* system. Activation of ChAT^+^ neurons by C21 administration resulted in more frequent migration of motor complexes (Figures 6G, 6I, and 6J; Table S4), whereas activation of TH^+^ neurons had no effect on CMMCs (Figure 6H; Table S4). The discrepancy between the *in vivo* and *ex vivo* results we observed may be due to activation of TH^+^ neurons in the sympathetic prevertebral ganglia that project to the gut.^30^ Overall, these data reveal that ChAT^+^ but not TH^+^ neurons in the gut mediate intestinal fluid balance and *ex vivo* colonic motility.

## DISCUSSION

While early pioneers of neuroscience in the 20^th^ century focused on the ENS as a model, more recent research has centered on the brain, and our understanding of the CNS has outpaced that of other neuronal systems in the body. As a result, basic knowledge of many aspects of neuronal architecture and function within the gut remains rudimentary.^15,21,22^ Here, using a viral delivery system with enhanced tropism for the ENS, we mapped the distribution of ChAT^+^ and TH^+^ neurons across the mouse GI tract and assayed the complex effects of their peripheral activation on physiology and function. Although the DREADD-based activation paradigm we use in this study is inherently artificial, the results reveal strikingly different roles for neuronal populations, with nearly every feature characterized (spatial distribution, metagenomic, metabolic, transcriptional, and proteomic profiles, and even physiological output) unique to cell type.

The viral vector we used, AAV-PHP.S, can transduce other neuronal subtypes in the PNS, such as those in the DRGs, and, with a strong ubiquitous promoter, induce transgene expression.^28^ To limit this off-target effect, we utilized a weaker promoter with increased ENS specificity and focused our analyses on GI tissue and lumen. By thus excluding most known extrinsic innervation pathways, we uncover cell-type-specific effects of gut-associated neuronal activation that are independent of signaling from the brain.

Exposure to the external environment charges the intestines with myriad responsibilities, including absorption and digestion of dietary nutrients, exclusion of xenobiotics, protection from enteric infection, and partnership with the gut microbiome. Deletion of ChAT in enteric neurons leads to microbiome dysbiosis,^62^ and we observed differences in the compositional profile (both metagenomic and proteomic) of the gut microbiome specific to activation of ChAT^+^ neurons. Notably, we found an expansion of Verrucomicrobia driven by *A. muciniphila*, which has been implicated in human diseases such as obesity,^63,64^ multiple sclerosis,^65,66^ and seizures.^67^ *A. muciniphila* metabolizes host-derived mucus as a nutrient source,^68^ consistent with the increase in luminal mucin proteins and digestive enzymes we observed in ChAT^+^-activated mice. A particularly interesting host-microbial interaction emerged from activation of TH^+^ cells, which led to a dramatic decrease in CHIA protein expression and a concomitant bloom in fungi, suggesting that neuronal circuits can regulate fungal populations in the gut. If validated, this intriguing host-microbial interaction could have implications for health. Our study does not, however, reveal the mechanism(s) by which ENS activation reshapes the gut microbial community structure, which may involve altered colonic motility, changes in mucus production, modulation of mucosal immune responses, and/or shifts in metabolism and nutrient availability.

The human gut microbiome possesses as much metabolic capacity as the liver; it is therefore no surprise that changes to both mouse gut physiology and the microbiome have major influences on the gut metabolome. In a striking example of mutualism, we report widespread changes to the pool of intestinal bile acids, molecules produced via host-microbial co-metabolism. Activation of ChAT^+^ neurons, but not TH^+^ neurons, impacted expression of NPC1L1, which is involved in cholesterol transport. In mammals, cholesterol is the substrate for production of primary bile acids, which are then metabolized exclusively by the gut microbiome into secondary bile acids. Bile acids play critical roles in fat absorption,^69^ gut motility,^70^ hormonal signaling^71^ and immune functions,^72^ and neurological conditions.^73^ Expression of bile salt hydrolases and hydratases increases the fitness of both commensal and pathogenic bacteria.^74–78^ While additional work is required to determine how the ENS affects the levels and constitution of the bile acid pool, understanding the processes that regulate synthesis of secondary bile acids may have implications for organ systems throughout the body.

Our study complements recent single-cell RNA sequencing (RNA-seq) studies of the ENS neuronal transcriptome^79^ by giving us the ability to selectively activate specific enteric neurons and explore the dynamic interplay between cells of various lineages in the gut. Importantly, the transcriptomic changes we observed may be a consequence of direct or indirect effects of neuronal activation. Indeed, induced activation of ChAT^+^ or TH^+^ neurons rapidly changed GI transit and fluid secretion patterns, which are only a fraction of the processes that may feed back on epithelial or immune cells, altering their gene expression profiles. Further single-cell analysis may help dissect the roles of the various intestinal cells that collaborate to coordinate gut functions.

The ENS adapts and responds to incredibly diverse molecular cues from the environment and must do so throughout the entire length and surface area of the intestines—the largest and most extensive internal organ with a rich network of neurons termed the “second brain”.^80^ Exposure to molecules from the diet or the microbiome may modulate ENS function, along with signals from outside the gut, such as the circulatory system. Curiously, many disorders of the brain are also associated with GI symptoms.^81–85^ While mechanisms linking the gut and the brain and their consequences for health are an active area of study, the impact of neuronal activation within the ENS has largely been unexplored. Here, we establish an experimental system that allows controlled activation of intrinsic and extrinsic neurons of the gut, separated from inputs from the brain, and demonstrate broad changes in the gut environment and its physiology that differ by activated neuronal population. The extensive datasets on activation of two major gut-associated neuronal populations that we generated should serve as a resource for further studies on the interconnected biological systems governing the complex relationship between gut physiology and the microbiome. Future deployment of this approach could enable mapping of neuronal connections into and out of the gut, providing insights into how the ENS networks with tissues throughout the body and advancing growing research into the many functions of the GI tract, an endeavor with important consequences for human health.

### Limitations of the study

It has been shown recently that a similar strategy using AAV-PHP.S-hSYN1 in ChAT-Cre mice also labels ChAT^+^ neurons in cardiac ganglia and that activation of cholinergic neurons reduces heart rate and blood pressure.^86,87^ Cardiac afferent neurons signal through the vagus nerve and jugular-nodose ganglia to the brain and in sensory pathways through the DRGs and spinal cord.^88^ Though there is no known direct route for signaling from cardiac ganglia to gut-associated neurons, it is possible that the gut may be impacted by an indirect route involving the CNS. In the future, further refinement of AAVs through directed evolution may generate serotypes with exclusive tropism for the ENS and allow full separation of the functions of intrinsic and extrinsic activation of gut-associated neuronal subsets. Since the hSYN1 promoter we used has been shown to drive expression only in neurons,^89,90^ we did not characterize other cell types, such as enteroendocrine cells (EECs), which have been shown recently to form synapses with enteric neurons and contribute to sensory transmission from the gut to the brain through the vagus nerve. Since these cells turn over every few days^91^ and have not been reported to express TH or ChAT, we think it unlikely that they are a major contributor to the activation-induced phenotypes we observed, but we cannot completely rule out their involvement.

## STAR★METHODS

### RESOURCE AVAILABILITY

Lead contact Further information and requests for resources and reagents should be directed to and will be fulfilled by the lead contact, Sarkis K. Mazmanian (sarkis@caltech.edu).

#### Materials availability

No new reagents were generated in this study.

#### Data and code availability

- Microbial sequencing data have been deposited at the European Bioinformatics Institute (ERP131523), Metabolomic data at UCSD MassIVE repository (MSV000084550), proteomic data at UCSD MassIVE repository (MSV000087917), and QuantSeq data at NCBI GEO repository (GSE180961) and are publicly available as of the date of publication. All other experimental data used to generate the figures reported in this paper can be found at (DOI:10.5281/zenodo.10525220, https://github.com/mazmanianlab/Griffiths_Yoo_et_al/) and are publicly available as of the date of publication.
- This paper does not report original code.
- Any additional information required to reanalyze the data reported in this paper is available from the lead contact upon request.

### EXPERIMENTAL MODEL AND STUDY PARTICIPANT DETAILS

#### Mice

All mouse experiments were performed in accordance with the NIH Guide for the Care and Use of Laboratory Animals using protocols approved by the Institutional Animal Care and Use Committee at the California Institute of Technology. Mice were fed *ad libitum* for the entire duration of experiments. Homozygous TH-Cre (gift to V.G. from Ted Ebendal, B6.129X1-Thtm1(cre)Te/Kieg^92^) and ChAT-Cre (Jackson Laboratories, Bar Harbor, ME- Stock# 028861, RRID:IMSR_JAX:028861) mice were bred to wild-type mice to yield the male and female heterozygous Cre-mice used for our studies. Wild-type specific pathogen free (SPF) C57BL/6 (Jackson Laboratories, Bar Harbor, ME- Stock #000664, RRID:IMSR_JAX:000664) males and females were used for breeding and experiments. Mice at 6–8 weeks of age were used for experiments.

#### Virus production

Virus was produced using the methods described in Challis et al., 2019^39^ and dx.doi.org/10.17504/protocols.io.bzn6p5he. Briefly, human embryonic kidney (HEK293T) cells were triple-transfected with pUCmini-iCAP-AAV-PHP.S, pHelper plasmid, and one of the following pAAV genomes: hSYN1-tdTomato, hSYN1-mRuby2, hSYN1-DIO-mRuby2, hSYN1-mNeonGreen, CAG-mNeonGreen, hSYN1-DIO-mNeonGreen, hSYN1-mTurquoise2, hSYN1-DIO-mTurquoise2, hSYN1-DIO-hM3Dq-mRuby2, CAG-GCaMP6f. Cells were grown in DMEM + Glutamax + Pyruvate (Gibco, Gaithersburg, MD- Stock# 10569–010) + 5% FBS + non-essential amino acids (Gibco, Gaithersburg, MD- Stock# 11140–050) + penicillin-streptomycin (Gibco, Gaithersburg, MD- Stock# 15070–063). Virus was precipitated from cells and supernatant with an 8% PEG solution (w/v), and purified by ultracentrifugation using 15%, 25%, 40%, 60% stacked iodixanol gradients.

### METHOD DETAILS

#### Systemic delivery of AAV

Mice were anesthetized using 2% isoflurane. Virus was titered to 1×10^12^ vg, resuspended in a volume of 100μLwith sterile PBS, and injected retro-orbitally as described in dx.doi.org/10.17504/protocols.io.bzn6p5he.

#### Neuronal activation of the GI tract

See dx.doi.org/10.17504/protocols.io.bzp5p5q6. TH-Cre and ChAT-Cre mice were used for these experiments. “Activated” mice were infected with AAV-PHP.S-hSYN1-DIO-hM3Dq-mRuby2 and “Control Mice” were infected with AAV-PHP.S-hSYN1-DIO-mRuby2. This was to control for both AAV-PHP.S-mediated expression and the effects of Compound 21 dihydrochloride (C21) (HelloBio, Princeton, NJ- HB6124). C21 was injected intraperitoneally (i.p.) at a dose of 3 mg/kg for 10 consecutive days to both groups of mice. Mice for timecourse experiments were single-housed in sterile cages with autoclaved water following the first C21 administration. Injections of C21 were administered at the same time every day (10a.m.). Mice were sacrificed 1 h after the day 10 injection.

#### Tissue preparation, immunohistochemistry, imaging, and quantification

Procedures are described in dx.doi.org/10.17504/protocols.io.bzp6p5re. 100 mg/kg of pentobarbital (Euthasol - Virbac, Carros, France) was administered i.p., and tissues were perfused with 30 mL of phosphate-buffered saline (PBS) and then cold 4% paraformaldehyde (PFA) in PBS. GI tract was post-fixed in 4% PFA overnight at 4°C, and stored in PBS +0.025% sodium azide. Tissues that underwent subsequent immunohistochemistry were made transparent by the passive CLARITY technique (PACT) as in Treweek et al., 2015.^33^ Briefly, perfused and fixed tissues were embedded with polymerized 4% (w/v) acrylamide, and lipids were eliminated using 8% (w/v) SDS solution. Jugular-nodose ganglia and dorsal root ganglia tissues were cryoprotected in 10% then 30% sucrose in PBS for 1 day each. Tissues were embedded and flash frozen in OCT and cryostat sectioned into 40 μM sections. Spinal cord and brain tissues were vibratome sectioned into 50 μM sections. Tissues were blocked in 3% donkey serum and permeabilized with PBS +0.3% Triton (PBST). Primary antibodies were incubated in PBST for 48 h and washed with PBST for 24 h (replacing the wash solution 3 times). Tissues were next incubated in secondary antibodies (and DAPI) for 24 h and washed in PBS for 48 h, intermittently replacing the wash solution with fresh PBS. Primary antibodies used were rabbit anti-PGP9.5 (1:300; Millipore Cat# AB1761-I, RRID:AB_2868444), rabbit anti-tyrosine hydroxylase (1:500, Abcam Cat# ab112, RRID:AB_297840), rabbit anti-choline acetyltransferase, (1:250, Abcam Cat# ab178850, RRID:AB_2721842), and mouse anti-NeuN (1:300, Abcam Cat# ab104224, RRID:AB_10711040). Secondary antibodies used were donkey anti-rabbit Alexa 568 (Thermo Fisher Scientific Cat# A10042, RRID:AB_2534017), goat anti-rabbit Alexa 647 (Thermo Fisher Scientific Cat# A-21245 (also A21245), RRID:AB_2535813), and goat anti-mouse Alexa 594 (Thermo Fisher Scientific Cat# A-11032, RRID:AB_2534091). GI tissues imaged for virally-expressed, endogenous fluorescence were made transparent using a sorbitol-based optical clearing method, Sca*l*eS as in Hama et al., 2015.^32^ Tissues were mounted in method-respective mounting media (RIMS and Scales S4) on a glass slide with a 0.5mm spacer (iSpacer, SunJin Lab Co.). Images were acquired on Zeiss LSM 780 or 880, and microscope, laser settings, contrast, and gamma remained constant across images that were directly compared. All confocal images were taken with the following objectives: Fluar 5 × 0.25 M27 Plan-Apochromat, 10 × 0.45 M27 (working distance 2.0 mm) and Plan-Apochromat 25 × 0.8 Imm Corr DIC M27 multiimmersion. Neurons in each ganglion were counted by counting cells that were of a distinct color. Colonic ganglia were defined as distinct if separated by a width of 3 or more neurons.

#### GCaMP6f fluorescence in *ex vivo* intestinal preparations

As described in dx.doi.org/10.17504/protocols.io.bzqap5se, small intestinal tissue was quickly harvested from ChAT-Cre mice, flushed, and placed in oxygenated (95% O_2_, 5% CO_2_), ice-cold Krebs-Henseleit solution for 1 h followed by 15 min at room temperature. A segment was cut along the mesenteric attachment and pinned flat (mucosa facing down) on a Sylgard-lined recording chamber (Warner Instruments, PH1) in oxygenated Krebs-Henseleit solution. C21 was added at 10nM and GCaMP6f fluorescence was detected on an upright microscope (Zeiss, Oberkochen, Germany- Examiner D1).

#### Metagenomics

Procedures are described in dx.doi.org/10.17504/protocols.io.bzqep5te.

#### Fecal collection

AAV-PHP.S-hSYN1-DIO-hM3Dq-mRuby2 (10^12^ vg) was delivered systemically to TH-Cre and ChAT-Cre mice. 3–4 week after infection, C21 (3 mg/kg) was administered daily for 10 consecutive days. Fecal pellets were collected in sterile containers one day before the initial C21 dose, and on day 2, 6, and 10 of injections.

#### Fecal sample DNA extraction and library preparation

DNA was extracted with the Qiagen MagAttract PowerSoil DNA kit as previously described in Marotz et al., 2017.^115^This protocol is optimized for an input quantity of 1 ng DNA per reaction. Prior to library preparation, input DNA was transferred to a 384-well plate and quantified using a PicoGreen fluorescence assay (ThermoFisher, Inc). Input DNA was then normalized to 1 ng in a volume of 3.5 μL of molecular-grade water using an Echo 550 acoustic liquid-handling robot (Labcyte, Inc). Enzyme mixes for fragmentation, end repair and A-tailing, ligation, and PCR were prepared and added in approximately 1:8 scale volumes using a Mosquito HV micropipetting robot (TTP Labtech). Fragmentation was performed at 37°C for 20 min, followed by end repair and A-tailing at 65°C for 30 min.

Sequencing adapters and barcode indices were added in two steps, following the iTru adapter protocol.^116^ Universal “stub” adapter molecules and ligase mix were first added to the end-repaired DNA using the Mosquito HV robot and ligation performed at 20°C for 1 h. Unligated adapters and adapter dimers were then removed using AMPure XP magnetic beads and a BlueCat purification robot (BlueCat Bio). 7.5 μL magnetic bead solution was added to the total adapter-ligated sample volume, washed twice with 70% EtOH, and then resuspended in 7 μL molecular-grade water.

Next, individual i7 and i5 were added to the adapter-ligated samples using the Echo 550 robot. Because this liquid handler individually addresses wells, and we used the full set of 384 unique error-correcting i7 and i5 indices, we were able to generate each plate of 384 libraries without repeating any barcodes, eliminating the problem of sequence misassignment due to barcode swapping.^117,118^ To ensure that libraries generated on different plates could be pooled if necessary, and to safeguard against the possibility of contamination due to sample carryover between runs, we also iterated the assignment of i7 to i5 indices each run, such that each unique i7:i5 index combination was repeated only once every 147,456 libraries. 4.5 μL of eluted bead-washed ligated samples was added to 5.5 μL of PCR master mix and PCR-amplified for 15 cycles. The amplified and indexed libraries were then purified again using magnetic beads and the BlueCat robot, resuspended in 10 μL water, and 9 μL of final purified library transferred to a 384-well plate using the Mosquito HV liquid-handling robot for library quantitation, sequencing, and storage. 384 samples were then normalized based on a PicoGreen fluorescence assay.

#### Shallow shotgun metagenome sequencing and diversity analysis

The Illumina data for each HiSeq lane was uploaded to Qiita, a tool with standardized pipelines for processing and analyzing metagenomic data.^119^ Adapter sequences were removed from the reads using the Atropos v.1.1.15 (RRID:SCR_023962, https://github.com/jdidion/atropos)^120^ command (from the qp-shogun 0.1.5 pipeline) and the trimmed sequences were downloaded from Qiita. The reads for each sample were filtered of any potential mouse contamination using Bowtie2 v.2–2.2.3^94^ (RRID:SCR_016368, https://bowtie-bio.sourceforge.net/bowtie2/index.shtml). The filtered reads were then aligned to the Web of Life (WoL) reference phylogeny^121^ with Bowtie2 using an adapted SHOGUN pipeline.^122^ The WoL contains 10,575 bacterial and archaeal genomes, with each genome representing an operational taxonomic unit (OTU). Sequencing reads that did not map to a single reference genome as well as reads that mapped to multiple genomes were not included in the analysis. If an OTU had a relative abundance less than 0.01% in a given sample, the OTU was not included for that sample. Additionally, OTUs with fewer than 5 assigned reads were not considered. The samples were rarefied to a depth of 12,750 reads and those with fewer than the rarefaction depth were excluded. The QIIME2 v.2019.7^44^ (RRID:SCR_021258, https://qiime2.org/) DEICODE plugin was used to calculate the Aitchison distances, a compositional beta diversity metric, and perform Robust Aitchison PCA to create biplots that visualize relationships between features and samples.^123^ The QIIME2 diversity plugin was used to calculate the other alpha- and beta-diversity metrics used in this study.

#### Metagenomics-based functional profiling

The filtered reads were also analyzed using HUMAnN2 v2.8.1^95^ (RRID:SCR_016280, https://huttenhower.sph.harvard.edu/humann2) to establish functional profiles for the samples. HUMAnN2 is a pipeline that begins by using MetaPhlAn2 to compile custom databases of reference genomes based on the species detected in a sample.^96^ HUMAnN2 then maps the filtered reads onto these custom databases and the reads that do not map to any of the references are then subjected to a translated search against UniProt Reference Clusters or UniRef^97^ (RRID:SCR_010646, https://www.uniprot.org/help/uniref). Here, the UniRef90 database was used for the translated search and installed according to the HUMAnN2 documentation. The results from both the search performed using the custom reference genome database and the search against the UniRef90 database were combined and the gene families identified in each sample were reported in units of reads per kilobase (RPKs) to account for gene length. HUMAnN2 also compared the gene families found in a sample with the MetaCyc pathways database^124^ (RRID:SCR_007778, https://metacyc.org/) and output a table reporting the pathway abundances found in each sample. After rarefying gene family tables to a depth of 166,000 RPKs and using a depth of 22,600 for pathway abundances, the QIIME2 diversity and DEICODE plugins were used to calculate alpha- and beta-diversity metrics. The metagenomics data from this study are available from. (https://github.com/mazmanianlab/Griffiths_Yoo_et_al/tree/main/metagenomics).

#### Metabolomics

Procedures are described in dx.doi.org/10.17504/protocols.io.bzqfp5tn.

#### Sample preparation

Frozen cecal samples were transported on dry ice for metabolomics analysis. The samples were weighed and an extraction solvent (1:1 methanol to water with an internal standard of 1 μM sulfamethazine) was added at a 1:10 mg to microliter ratio. The samples were then homogenized using a TissueLyser II (Qiagen) for 5 min at 25 Hz followed by a 15 min centrifugation at 14,000 rpm. 120 μL of supernatant was transferred to a 96-well DeepWell plate (Eppendorf) and lyophilized using a CentriVap Benchtop Vacuum Concentrator (Labconco) and stored at −80°C. At the time of data acquisition, the lyophilized plates were resuspended in a 1:1 methanol to water solvent spiked with 1 μM of sulfadimethoxine. The plates were vortexed for 2 min, centrifuged at 14,000 rpm for 15 min and 120 μL of the supernatant was transferred to a 96-well autosampler plate (Eppendorf). Plates were stored at 4°C prior to LCMS analysis.

#### Data acquisition

The untargeted metabolomics analysis was completed using an ultra-high performance liquid chromatography system (Thermo Dionex Ultimate 3000 UHPLC) coupled to an ultra-high resolution quadrupole time of flight (qTOF) mass spectrometer (Bruker Daltonics MaXis HD). A Phenomenex Kinetex column (C18 1.7 μm, 2.1 mm × 50 mm) was used for chromatographic separation. An injection volume of 5 μL was used for each sample and a flow-rate of 0.500 mL was used throughout the analysis. The mobile phase consisted of solvent A: 100% LC-MS grade water spiked with 0.1% formic acid and solvent B: 100% LC-MS grade acetonitrile spiked with 0.1% formic acid. The chromatographic gradient was as follows: 0.0–1.0 min, 5% B; 1.0–9.0 min, 5–100% B; 9.0–11.0 min, 100% B; 11.0–11.5 min, 100–5% B; 11.5–12.5 min, 5% B. Data was collected with electrospray ionization in positive mode, and was saved as.d file folders.

#### Data processing

The raw.d data files were converted to mzXML format using Bruker Compass DataAnalysis 4.1 software. The resulting.mzXML file, the original.d file folders, and basic prep information sheet are stored in the UC San Diego MassIVE data repository under the accession number MSV000084550. For MS1 level feature detection, the open-source software MZmine version 2.51 (RRID:SCR_012040, https://mzmine.github.io/) was used. The parameters used are as follows: 1) Mass Detection (Centroid, Noise Level MS1 1E3, MS2 1E2); 2) ADAP Chromatogram Builder (Min Group size in # of scans = 3, Group Intensity Threshold = 3E3, Min Highest Intensity = 1E3, m/z tolerance 0.01 m/z or 10.0 ppm); 3) Chromatogram Deconvolution (Local Minimum Search>Chromatographic Threshold 0.01%, Minimum in RT range 0.50 min, <Minimum Relative Height 0.01%, Minimum Absolute Height 3E3, Min Ratio of Peak Top/Edge 2, Peak Duration Range 0.05–0.50 min; m/z Calculation Auto, m/z range for MS2 pairing 0.01 Da, and RT Range for MS2 Pairing 0.1 min); Isotopic Peaks Grouper (m/z Tolerance 0.01 m/z or 10.0 ppm, Retention Time Tolerance 0.3 min, Maximum Charge 4, Representative Ion Most Intense); Join Aligner (m/z Tolerance 0.01 m/z or 10.0 ppm, Weight for m/z 75, Retention Time Tolerance 0.3 min, Weight for RT 25); Gap-Filling Peak Finder (Intensity Tolerance 20%, m/z Tolerance 0.005 m/z or 10.0 ppm, Retention Time Tolerance 0.2 min). The resulting feature table was saved as a.csv file and.mgf file for use in GNPS and MetaboAnalyst.

#### Molecular networking and statistical analysis

Molecular networking was performed using the feature networking tool available through the Global Natural Products Social Molecular Networking (GNPS, RRID:SCR_019012, https://gnps.ucsd.edu/ProteoSAFe/static/gnps-splash.jsp) portal:https://gnps.ucsd.edu/ProteoSAFe/index.jsp?params=%7B%22workflow%22:%22FEATURE-BASED-MOLECULAR-NETWORKING%22,%22library_on_server%22:%22d.speclibs;%22%7D.

The annotations obtained using this workflow fall under MSI level 2 or 3 and were used for feature analysis.^46^ Briefly, level 2 compounds are putatively annotated, meaning they are not identified using chemical reference standards but rather based on physical properties and/or spectral similarities to available spectral libraries (publicly available and purchased NIST17 CID). Level 3 compounds are putatively characterized classes of compounds identified similarly to level 2 compounds. The feature-based molecular networking workflow on GNPS^125^ was utilized in order to analyze the spectra associated with the feature tables produced using the open source software Mzmine version 2.51^98^ (RRID:SCR_012040, https://mzmine.github.io/). The.mgf and.csv outputs from MZmine v2.51 were used to run the workflow. The GNPS workflow parameters used were as follows: Precursor Ion Mass 0.02 Da, Fragment Ion Mass Tolerance 0.02 Da, Min Pairs Cos 0.7, Minimum Matched Fragments 6, Maximum Shift Between Precursors 500 Da, Network TopK 10, Maximum Connected Component Size (Beta) 100, and the files were row sum normalized. Default parameters were used for the rest of the settings. Visualizations and statistical analyses were performed using QIIME 2 v.2019.10^44^ (RRID:SCR_021258), MetaboAnalyst^99^ (RRID:SCR_015539, https://www.metaboanalyst.ca/) and Cytoscape v3.7.2^100^ (RRID:SCR_003032, https://cytoscape.org/). The metabolomics data from this study are available from. (https://github.com/mazmanianlab/Griffiths_Yoo_et_al/tree/main/metabolomics).

#### Proteome preparation

Procedures are described in dx.doi.org/10.17504/protocols.io.bzqcp5sw.

#### Protein extraction

Mice were sacrificed 1 h after the final C21 administration and cecal contents were isolated and resuspended in 400 μL of phosphate buffered solution and centrifuged at 20,000 xg to spin down cells and lysate. Protein was isolated from the resulting supernatant using Wessel-Flügge’s methanol/chloroform extraction method (Wessel and Flügge, 1984). Briefly, MeOH and chloroform were added to the samples at a 4:1 and 1:1 ratio, respectively. Next, dH_2_O was added at a 3:1 ratio, samples were vortexed and centrifuged at 20,000 xg. Resulting precipitated protein was collected and washed with MeOH. Precipitated protein was centrifuged and left to air dry, and stored at −20°C until protein digestion.

#### In-solution protein digestion and desalting

Precipitated protein samples were denatured in 40 μL of 8M Urea (100 mM Tris-HCl pH 8.5). To reduce disulfide bonds, 1.25 μL of 100 mM Tris(2-carboxyethyl)Phosphine was added and incubated at room temperature (RT) for 20 min. Then 1.8 μL of 250 mM iodoacetamide was added and incubated at RT in the dark to alkylate cysteines. The first step of digestion was initiated by adding 1 μL of 0.1 μg/μL of lysyl endopeptidase. After 4 h of incubation, the urea concentration was adjusted to 2 M by adding 120 μL of 100 mM Tris-HCl pH 8.5. The second step of digestion was done by adding 2.5 μL of 2μg/μL trypsin plus 1.6 μL of 100 mM CaCl_2_ and incubating overnight in the dark. Formic acid was added to stop trypsin digestion. Digested peptides were desalted by HPLC using a C8 peptide microtrap (Microm Bioresources), lyophilized, and diluted to 200 ng/μL in 0.2% formic acid prior to LC-MS/MS analysis.

#### LC-MS/MS

Samples were analyzed on a Q Exactive HF Orbitrap mass spectrometer coupled to an EASY nLC 1200 liquid chromatographic system (Thermo Scientific, San Jose, CA). Approximately 200 ng of peptides were loaded on a 50 μm I.D. × 25 cm column with a 10 μm electrospray tip (PicoFrit from New Objective, Woburn, MA) in-house-packed with ReproSil-Pur C18-AQ 1.9 μm (Dr. Maisch, Ammerbuch, Germany). Solvent A consisted of 2% MeCN in 0.2% FA and solvent B consisted of 80% MeCN in 0.2% FA. A non-linear 60 min gradient from 2% B to 40% B was used to separate the peptides for analysis. The mass spectrometer was operated in a data-dependent mode, with MS1 scans collected from 400 to 1650 m/z at 60,000 resolution and MS/MS scans collected from 200 to 2000 m/z at 30,000 resolution. Dynamic exclusion of 45 s was used. The top 12 most abundant peptides with charge states between 2 and 5 were selected for fragmentation with normalized collision energy of 28.

#### Peptide and protein identification

Thermo.raw files were converted to.ms1 and.ms2 files using RawConverter 1.1.0.18 operating in data dependent mode and selecting for monoisotopic m/z. Tandem mass spectra (.ms2 files) were identified by a database search method using the Integrated Proteomics Pipeline 6.5.4 (IP2, Integrated Proteomics Applications, Inc., http://www.integratedproteomics.com). Briefly, databases containing forward and reverse (decoy)^126,127^ peptide sequences were generated from *in silico* trypsin digestion of either the mouse proteome (UniProt; Oct. 2, 2019)^101^ or protein sequences derived from large comprehensive public repositories (ComPIL 2.0).^102^ Tandem mass spectra were matched to peptide sequences using the ProLuCID/SEQUEST (1.4)^103,104^ software package. The validity of spectrum-peptide matches was assessed using the SEQUEST-defined parameters XCorr (cross-correlation score) and DeltaCN (normalized difference in cross-correlation scores) in the DTASelect2 (2.1.4)^105,106^ software package. Search settings were configured as follows: (1) 5 ppm precursor ion mass tolerance, (2) 10 ppm fragment ion mass tolerance, (3) 1% peptide false discovery rate, (4) 2 peptide per protein minimum, (5) 600–6000 Da precursor mass window, (6) 2 differential modifications per peptide maximum (methionine oxidation: M+15.994915 Da), (7) unlimited static modifications per peptide (cysteine carbamidomethylation: C+57.02146 Da), and (8) the search space included half- and fully-tryptic (cleavage C-terminal to K and R residues) peptide candidates with unlimited (mouse database, custom metagenomic shotgun database) or 2 missed cleavage events (ComPIL 2.0).

#### Differential analysis of detected proteins using peptide-spectrum matches (spectral counts)

Detected proteins were grouped by sequence similarity into “clusters” using CD-HIT 4.8.1 (RRID:SCR_007105, http://weizhong-lab.ucsd.edu/cd-hit/)^107,108,128^ at the following similarity cut-offs: 65%, 75%, 85%, and 95%. The following is an example command line input: “cd-hit -i fastafile.fasta -o outputfile -c 0.65 -g 1 -d 0”. Tandem mass spectra identified as peptides (peptide spectrum matches, PSMs) were mapped to CD-HIT generated clusters. PSMs mapping to >1 cluster were discarded. Cluster-PSM tables were generated and differential analysis was performed in DESeq2 (1.25.13, RRID:SCR_015687, https://bioconductor.org/packages/release/bioc/html/DESeq2.html).^109^ Briefly, count data (PSMs) were modeled using the negative binomial distribution, and the mean-variance relationship was estimated. Variance was estimated using an information sharing approach whereby a single feature’s (or cluster’s) variance was estimated by taking into account variances of other clusters measured in the same experiment. Feature significance calling and ranking were performed using estimated effect sizes. Multiple testing correction was performed by the Benjamini-Hochberg method within the DESeq2 package. Volcano plots were generated in Prism (GraphPad).

#### Differential analysis of detected proteins using ion intensity (precursor intensity)

Detected proteins were grouped into “clusters” by sequence similarity using CD-HIT 4.8.1^107,108,128^ at the following similarity cutoffs: 65%, 75%, 85%, and 95%. The following is an example command line input: “cd-hit −i fastafile.fasta −o outputfile −c 0.65 −g 1 −d 0”. Using the Census software package^129^ (Integrated Proteomics Pipeline 6.5.4), peptide ion intensities were calculated from.ms1 files. Peptide ion intensities were assigned to their parent peptide, then parent peptides were mapped to their appropriate CD-HIT generated clusters. Ion intensities belonging to parent peptides that mapped to >1 CD-HIT cluster were discarded. Clusterion intensity tables were generated.

Ion intensity data were analyzed using the Differential Enrichment analysis of Proteomics data DEP package (RRID:SCR_023090, https://bioconductor.org/packages/release/bioc/html/DEP.html)^110^ operating in R. Intensity values were automatically Log2 transformed in DEP. The cluster list was subsequently filtered with the ‘filter_proteins’ function such that clusters with missing values above a 65% threshold were discarded. Remaining intensities were further transformed by the ‘normalize_vsn’ function.^130^ Missing data in remaining clusters were imputed using a mixed approach. Clusters where either the control or treatment group contained only null entries were classified as ‘missing not at random’ (MNAR) and imputed with 0 values. All other groups were treated as ‘missing at random’ (MAR) and imputed using the maximum likelihood method (‘MLE’).^131^ Note that for a given cluster, missing values for treatment groups were imputed separately by treatment group. Differential expression analyses were performed on filled-in cluster-ion intensity tables using the ‘test_diff’ function^132^ and multiple testing correction was performed using the ‘add_rejections’ function.

#### Network analysis using the STRING database

Upregulated proteins with a nominal p value <0.2 were searched against protein-protein interactions in the STRING database^111^ (RRID:SCR_005223, http://www.string-db.org) where high confidence interactions are selected for. Briefly, the STRING database sources protein-protein interactions from primary databases consisting of genomic context predictions, high-throughput lab experiments, (conserved) co-expression, automated text mining, and previous knowledge in databases.^111^

#### Metaproteome analysis using unipept

Upregulated tryptic, microbial peptide sequences, with fold change and nominal p value cutoffs of >2 and <0.2, respectively, were input into Unipept (http://unipept.ugent.be),^133,134^ equating leucine and isoleucine and filtering duplicate peptides. Briefly, Unipept indexes tryptic peptide sequences from the UniProtKB and details peptides with NCBI’s taxonomic database. Lowest common ancestor was calculated for each tryptic peptide. The proteomics data from this study are available from. (https://github.com/mazmanianlab/Griffiths_Yoo_et_al/tree/main/proteomics).

#### 3′ mRNA sequencing

Procedures are described in dx.doi.org/10.17504/protocols.io.bzqbp5sn.

#### Tissue collection and RNA extraction

Mice were cervically dislocated and the GI tract was removed. 1 cm of tissue above and below the cecum were dissected and cleaned to represent tissue from the distal SI and proximal colon, respectively. Tissue was homogenized in TRIzol (ThermoFisher Scientific, Waltham. MA- Cat. No. 15596018) solution using a bead-based homogenizing method, and total RNA was extracted using chloroform per manufacturer’s instructions.

#### Library preparation, sequencing, and analysis

The cDNA libraries were prepared using the QuantSeq 3′mRNA-Seq Library Prep Kit FWD for Illumina (Lexogen, Greenland, NH) supplemented with UMI (unique molecular index) as per the manufacturer’s instructions. Briefly, total RNA was reverse transcribed using oligo (dT) primers. The second cDNA strand was synthesized by random priming, in a manner such that DNA polymerase is efficiently stopped when reaching the next hybridized random primer, so only the fragment closest to the 3′ end gets captured for later indexed adapter ligation and PCR amplification. UMIs were incorporated to the first 6 bases of each read, followed by 4 bases of spacer sequences. UMIs are used to eliminate possible PCR duplicates in sequencing datasets and therefore facilitate unbiased gene expression profiling. The basic principle behind the UMI deduplication step is to collapse reads with identical mapping coordinates and UMI sequences. This step helps increase the accuracy of sequencing read counts for downstream analysis of gene expression levels. The processed libraries were assessed for size distribution and concentration using the Bioanalyzer High Sensitivity DNA Kit (Agilent Technologies, Santa Clara, CA- Cat. No. 5067–4626 and −4627). Pooled libraries were diluted to 2 nM in EB buffer (Qiagen, Hilden, Germany, Cat. No. 19086) and then denatured using the Illumina protocol. The libraries were pooled and diluted to 2 nM using 10 mM Tris-HCl, pH 8.5 and then denatured using the Illumina protocol. The denatured libraries were diluted to 10 p.m. with pre-chilled hybridization buffer and loaded onto an Illumina MiSeq v3 flow cell for 150 cycles using a single-read recipe according to the manufacturer’s instructions. Single-end 75 bp reads (max 4.5M reads) were obtained. De-multiplexed sequencing reads were generated using Illumina BaseSpace.

UMI specific workflows that were developed and distributed by Lexogen were used to extract reads that are free from PCR artifacts (i.e., deduplication). First, the umi2index tool was used to add the 6 nucleotide UMI sequence to the identifier of each read and trim the UMI from the start of each read. This generated a new FASTQ file, which was then processed through trimming and alignment. Second, after the quality and polyA trimming by BBDuk^112^ (Bestus Bioinformaticus Duk, RRID:SCR_016969, https://jgi.doe.gov/data-and-tools/bbtools/bb-tools-user-guide/bbduk-guide/) and alignment by HISAT2 (version 2.1.0, RRID:SCR_015530, https://daehwankimlab.github.io/hisat2/),^113^ the mapped reads were collapsed according to the UMI sequence of each read. Reads were collapsed if they had the same mapping coordinates (CIGAR string) and identical UMI sequences. Collapsing reads in this manner removes PCR duplicates. Read counts were calculated using HTSeq (RRID:SCR_005514, https://htseq.readthedocs.io/en/release_0.9.1/)^114^ by supplementing Ensembl gene annotation (GRCm38.78). Raw read counts were run through ShinySeq to obtain differentially expressed genes and downstream gene ontology analyses.^135^ The RNAseq data from this study are available from. (https://github.com/mazmanianlab/Griffiths_Yoo_et_al/tree/main/RNAseq).

#### Whole gut transit time, fecal water content, and fecal output

Procedures are described in dx.doi.org/10.17504/protocols.io.36wgq3p1xlk5/v1.

6% (w/v) carmine red (Sigma-Aldrich, St. Louis, MO) with 0.5% methylcellulose (Sigma-Aldrich) was dissolved and autoclaved prior to use. ChAT-Cre and TH-Cre mice were administered C21 (3 mg/kg) intraperitoneally, and subsequently orally gavaged with 150 μL of carmine red solution. Mice were single-housed with no bedding for the duration of the experiment, and animals were not fasted beforehand. Over the 5 h following gavage, the time of expulsion was recorded for each fecal pellet, and each pellet was collected in pre-weighed, 1.5 mL microcentrifuge tube. Each pellet collected was checked for the presence of carmine red, and the time of initial carmine red pellet expulsion was recorded as GI transit time. The mass of collected fecal pellets was determined, and pellets were left to dry in an 80°C oven for 2 days before weighing the desiccated pellets and calculating the pellets’ initial water content. Fecal output rate for each mouse was calculated as the total number of pellets expelled during the 5 h time course post-C21 administration divided by the time the last fecal pellet was expelled.

#### Colonic migrating motor complexes in *ex vivo* intestinal preparations

Procedures are described in dx.doi.org/10.17504/protocols.io.n92ldm61nL5b/v1.

Intact colons were dissected from cervically-dislocated ChAT-Cre and TH-Cre mice, flushed and placed in pre-oxygenated (95% O_2_, 5% CO_2_) Krebs-Henseleit solution at RT. Proximal and distal ends were cannulated to 2 mm diameter tubes and secured in the center of an organ bath with continuously oxygenated Krebs-Henseleit solution at 37°C. Syringe pumps were connected to the inlet and outlet tubes to maintain a flow of solution at a rate of 500 μL/min through the colon. The system was allowed to equilibrate for 30 min before recording. Baseline recordings were taken for 30 min, then the Krebs solution in the organ bath was briefly removed, mixed with C21 to a final concentration of 2 μM, and replaced in the organ bath. Recordings were taken for another 30 min.

### QUANTIFICATION AND STATISTICAL ANALYSIS

Statistical methodologies and software used for performing various types of multi-omic analysis in this work are cited where appropriate in the STAR Methods text. The p value calculations for viral transduction, microbiome differences, gastrointestinal function, and animal welfare were done using Prism GraphPad v.9.2.0. The specific statistical test used for each figure is denoted in the figure legends. Error bars represent the standard error of the mean unless otherwise stated.

## Supplementary Material

1

2

3

4

5

6

## Figures and Tables

**Table:** KEY RESOURCES TABLE

REAGENT or RESOURCE	SOURCE	IDENTIFIER
Antibodies		
rabbit anti-PGP9.5	Millipore	Cat# AB1761-I, RRID:AB_2868444
rabbit anti-tyrosine hydroxylase	Abcam	Cat# ab112, RRID:AB_297840
rabbit anti-choline acetyltransferase	Abcam	Cat# ab178850, RRID:AB_2721842
mouse anti-NeuN	Abcam	Cat# ab104224, RRID:AB_10711040
donkey anti-rabbit Alexa 568	Thermo Fisher Scientific	Cat# A10042, RRID:AB_2534017
goat anti-rabbit Alexa 647	Thermo Fisher Scientific	Cat# A-21245, RRID:AB_2535813
goat anti-mouse Alexa 594	Thermo Fisher Scientific	Cat# A-11032, RRID:AB_2534091
Chemicals, peptides, and recombinant proteins		
Compound 21 dihydrochloride (C21)	HelloBio	Cat# HB6124
Critical commercial assays		
MagAttract PowerSoil DNA kit	Qiagen	Cat# 27100–4-EP
PicoGreen fluorescence assay	ThermoFisher	Cat# P7589
QuantSeq 3′mRNA-Seq Library Prep Kit FWD for Illumina	Lexogen	Cat# 015
Bioanalyzer High Sensitivity DNA Kit	Agilent Technologies	Cat# 5067–4626 and −4627
Deposited data		
Metagenomic data	This paper	EBI accession: ERP131523
Metabolomic data	This paper	UCSD MassIVE: MSV000084550
Proteomic data	This paper	UCSD MassIVE: MSV000087917
QuantSeq data	This paper	GEO: GSE180961
Experimental models: Cell lines		
HEK293T/17	ATCC	Cat# CRL-11268, RRID:CVCL_1926
Experimental models: Organisms/strains		
Mouse: B6.129X1-Thtm1(cre)Te/Kieg (TH-cre)	Gift from Ted Ebendal^92^	RRID:MGI:3487234
Mouse: B6J.129S6-Chattm2(cre)Lowl/MwarJ (ChAT-cre)	Jackson Laboratories	ME- Stock# 028861, RRID:IMSR_JAX:028861
Mouse: C57BL/6	Jackson Laboratories	ME- Stock# 000664, RRID:IMSR_JAX:000664
Recombinant DNA		
Plasmid: AAV-PHP.S	Gradinaru Lab	Addgene plasmid# 103006, RRID:Addgene_103006
Plasmid: hSYN1-tdTomato	This study	Adapted from Addgene plasmid# 99116, RRID: Addgene_99116 and plasmid# 99126, RRID:Addgene_99126
Plasmid: hSYN1-mRuby2	Gradinaru Lab	Addgene plasmid# 99126, RRID:Addgene_99126
Plasmid: hSYN1-DIO-mRuby2	This study	Adapted from Addgene plasmid# 99126, RRID:Addgene_99126
Plasmid: CAG-mNeonGreen	Gradinaru Lab	Addgene plasmid# 99134, RRID:Addgene_99134
Plasmid: hSYN1-mNeonGreen	Gradinaru Lab	Addgene plasmid# 99135, RRID:Addgene_99135
Plasmid: hSYN1-DIO-mNeonGreen	This study	Adapted from Addgene plasmid# 99125, RRID:Addgene_99135
Plasmid: hSYN1-mTurquoise2	Gradinaru Lab	Addgene plasmid# 99125, RRID: Addgene_99125
Plasmid: hSYN1-DIO-mTurquoise2	This study	Adapted from Addgene plasmid# 99125, RRID: Addgene_99125
Plasmid: hSYN1-DIO-hM3Dq-mRuby2	This study	Adapted from Addgene plasmid# 50474 RRID:Addgene_50474 and Addgene plasmid# 99126, RRID:Addgene_99126
Plasmid: CAG-GCaMP6f	This study	Adapted from Addgene plasmid# 100837 RRID:Addgene_100837
Software and algorithms		
FIJI	Schindelin et al.^93^	RRID:SCR_002285, https://imagej.net/software/fiji
R	R Project for Statistical Computing	RRID:SCR_001905, https://www.r-project.org/
Qiita	Bolyen et al.^44^	https://www.qiime2.org/
Bowtie2	Langmead and Salzberg^94^	RRID:SCR_016368, http://bowtie-bio.sourceforge.net/bowtie2/index.shtml
HUMAnN2 v2.8.1	Franzosa et al.^95^	RRID:SCR_016280, https://huttenhower.sph.harvard.edu/humann2
QIIME2 v.2019.10	Bolyen et al.^44^	RRID:SCR_021258
MetaPhlAn2	Truong et al.^96^	RRID:SCR_004915, https://huttenhower.sph.harvard.edu/metaphlan2
UniRef	Suzek et al.^97^	RRID:SCR_010646, https://www.uniprot.org/help/uniref
Mzmine version 2.51	Pluskal et al.^98^	RRID:SCR_012040, https://mzmine.github.io/
MetaboAnalyst	Xia et al.^99^	RRID:SCR_015539, https://www.metaboanalyst.ca/
Cytoscape v3.7.2	Shannon et al.^100^	RRID:SCR_003032, https://cytoscape.org/
Global Natural Products Social Molecular Networking (GNPS)	Wang et al.^45^	RRID:SCR_019012, https://gnps.ucsd.edu/ProteoSAFe/static/gnps-splash.jsp
UniProt	The Uniprot Consortium^101^	RRID: SCR_002380, https://www.uniprot.org/
ComPIL 2.0	Park et al.^102^	https://github.com/robinparky/prolucidComPIL
ProLuCID/SEQUEST (1.4)	Xu et al.^103,104^	http://fields.scripps.edu/yates
DTASelect2 (2.1.4)	Cociorva et al.^105^ & Tabb et al.^106^	http://fields.scripps.edu/yates
CD-HIT 4.8.1	Fu et al.^107^ & Li et al.^108^	RRID:SCR_007105, http://weizhong-lab.ucsd.edu/cd-hit/)
DESeq2	Love et al.^109^	1.25.13, RRID:SCR_015687, https://bioconductor.org/packages/release/bioc/html/DESeq2.html
Differential Enrichment analysis of Proteomics data DEP package	Zhang et al.^110^	RRID:SCR_023090, https://bioconductor.org/packages/release/bioc/html/DEP.html
STRING database	Szklarczyk et al.^111^	RRID:SCR_005223, https://www.string-db.org/
BBDuk (BBTools)	Bushnell et al.^112^	Bestus Bioinformaticus Duk, RRID:SCR_016969, https://jgi.doe.gov/data-and-tools/bbtools/bb-tools-user-guide/bbduk-guide/
HISAT2	Kim et al.^113^	version 2.1.0, RRID:SCR_015530, https://daehwankimlab.github.io/hisat2/
HTSeq	Anders et al.^114^	RRID:SCR_005514, https://htseq.readthedocs.io/en/release_0.9.1/)
GraphPad Prism v9.2.0	GraphPad Software	RRID:SCR_002798, https://www.graphpad.com/

## Highlights

- ChAT^+^ and TH^+^ neurons show distinct spatial organization within the ENS of mice
- ChAT^+^ and TH^+^ neurons differentially shape the composition of the gut microbiome
- Gut-associated TH^+^ neurons regulate fungal colonization of the gut
- Gut-associated ChAT^+^ neuron activation promotes fluid secretion and colonic motility
